# Phospholipase A2 group IVD mediates the transacylation of glycerophospholipids and acylglycerols

**DOI:** 10.1016/j.jlr.2024.100685

**Published:** 2024-10-25

**Authors:** Johannes Breithofer, Dominik Bulfon, Nermeen Fawzy, Martin Tischitz, Clara Zitta, Lennart Hartig, Gernot F. Grabner, Anita Pirchheim, Hubert Hackl, Ulrike Taschler, Achim Lass, Carmen Tam-Amersdorfer, Herbert Strobl, Dagmar Kratky, Robert Zimmermann

**Affiliations:** 1 Institute of Molecular Biosciences, University of Graz, Graz, Austria; 2 Gottfried Schatz Research Center, Molecular Biology and Biochemistry, Medical University of Graz, Graz, Austria; 3 Institute of Bioinformatics, Biocenter, Medical University of Innsbruck, Innsbruck, Austria; 4 BioTechMed-Graz, Graz, Austria; 5 Field of Excellence BioHealth, University of Graz, Graz, Austria; 6 Otto Loewi Research Center for Vascular Biology, Immunology and Inflammation, Division of Immunology, Medical University of Graz, Graz, Austria

**Keywords:** PLA2G4D, cPLA2δ, transacylase, monoacylglycerol, diacylglycerol, glycerolipids, phospholipases, phospholipids/metabolism, triacylglycerol, enzymolgy/enzyme mechanisms

## Abstract

In mammalian cells, glycerolipids are mainly synthesized using acyl-CoA–dependent mechanisms. The acyl-CoA–independent transfer of fatty acids between lipids, designated as transacylation reaction, represents an additional mechanism for lipid remodeling and synthesis pathways. Here, we demonstrate that human and mouse phospholipase A2 group IVD (PLA2G4D) catalyzes transacylase reactions using both phospholipids and acylglycerols as substrates. In the presence of monoglycerol and diacylglycerol (MAG and DAG), purified PLA2G4D generates DAG and triacylglycerol, respectively. The enzyme also transfers fatty acids between phospholipids and from phospholipids to acylglycerols. Overexpression of PLA2G4D in COS7 cells enhances the incorporation of polyunsaturated fatty acids into triacylglycerol stores and induces the accumulation of lysophospholipids. In the presence of exogenously added MAG, the enzyme strongly increases cellular DAG formation, while MAG levels are decreased. PLA2G4D is not or poorly detectable in commonly used cell lines. It is expressed in keratinocytes, where it is strongly upregulated by proinflammatory cytokines. *Pla2g4d*-deficient mouse keratinocytes exhibit complex lipidomic changes in response to cytokine treatment, indicating that PLA2G4D is involved in the remodeling of the lipidome under inflammatory conditions. Transcriptomic analysis revealed that PLA2G4D modulates fundamental biological processes including cell proliferation, differentiation, and signaling. Together, our observations demonstrate that PLA2G4D has broad substrate specificity for fatty acid donor and acceptor lipids, allowing the acyl-CoA-independent synthesis of both phospholipids and acylglycerols. Loss-of-function studies indicate that PLA2G4D affects metabolic and signaling pathways in keratinocytes, which is associated with complex lipidomic and transcriptomic alterations.

Lipid hydrolases are active in all body compartments, where they play essential roles in lipid degradation, transport, and remodeling. Many (phospho)lipases are serine hydrolases characterized by a conserved catalytic dyad or triad (1). The triad typically includes a nucleophilic serine residue embedded in a GXSXG motif, an acidic aspartate or glutamate, and a histidine (2, 3). The dyad is composed of the GXSXG motif and an aspartate (4). The catalytic reaction starts with the binding of the lipid substrate and the nucleophilic attack of the serine on the carbonyl carbon atom of the ester bond. This leads to the cleavage of the substrate, the release of the alcohol product, and to the formation of an acyl-enzyme intermediate, where the substrate-derived FA is covalently bound to the active serine. The next step is the deacylation of the acyl-enzyme intermediate, in which a water molecule hydrolyzes the covalent bond, releasing the FA and regenerating the enzyme. Alternatively, the acyl-enzyme intermediate reacts with an alcohol instead of water leading to the formation of an ester bond and enzyme regeneration, a process termed alcoholysis. In cases where the acyl acceptor is a lipid, this mechanism enables transacylase reactions that catalyze the transfer of FAs between different lipid molecules (5).

Transacylase reactions are well known to catalyze the synthesis of complex lipids. For example, lecithin:cholesterol acyltransferase converts free cholesterol of circulating lipoproteins into cholesterol ester, which is then sequestered into the core of lipoprotein particles (6). The microsomal enzyme lecithin:retinol acyltransferase catalyzes the transacylation of all-trans-retinol into all-trans-retinyl ester and critically affects the formation of retinyl ester (the storage form of vitamin A) in hepatic stellate cells and retinal pigment epithelium (7). Phospholipase A2 group IV (PLA2G4) E acylates phosphatidylethanolamine (PE), leading to the formation of N-acyl PE, the precursor of the endocannabinoid N-arachidonoyl ethanolamine (anandamide) and other N-acyl ethanolamines (8, 9). The transacylase patatin-like phospholipase domain-containing protein 1 (PNPLA1) catalyzes the biosynthesis of ω-O-acylceramides, which are essential for a functional skin barrier (10). Recent observations also suggest that transacylases are required for the synthesis of the late endosomal/lysosomal lipid bis(monoacylglycero)phosphate (11, 12). Overall, published data suggests that transacylation reactions contribute to the synthesis of structural, storage, and bioactive lipids.

In our study, we identified PLA2G4D as a cytosolic lipid hydrolase and transacylase. The enzyme is highly expressed in keratinocytes and can mediate transacylation reactions within and between phospholipids and acylglycerols.

## Materials and methods

### Cell culture

COS7 (CRL-1651, ATCC) and HaCaT (CVCL_0038, CLS) cells were cultured in DMEM 4.5 g/L glucose (Gibco, Thermo Fisher Scientific, Waltham) supplemented with 10% FBS as well as 100 IU/ml penicillin and 100 IU/ml streptomycin (DMEM+/+). Expi29F cells (A14527, Gibco) were cultured in Expi293T Expression Medium (A1435101, Gibco) in 25 ml or 10 ml culture volumes using 125 ml vented Erlenmeyer flasks (431143, Corning, NY) or 50 ml CELLSTAR cell reactor tubes (227245, Greiner Bio-One, Kremsmünster, Austria) on an orbital shaker platform at 125 or 220 rpm, respectively. Cell lines were regularly checked for mycoplasma contamination using the Mycoplasmacheck Service from Eurofins Genomics GmbH (Germany). All cells were grown under standard conditions (37°C; 95% humidified atmosphere; 7% CO_2_).

### Transfection of COS7 cells

For transient protein expression, 2 × 10^5^ or 1.2 × 10^6^ COS7 cells were seeded into 6-well plates or 100 mm culture dishes, respectively. The next day, cells were transfected with the Lipofectamine 3000 transfection reagent (L3000001, Invitrogen, Waltham). One well of a 6-well plate was transfected according to the following protocol: 125 μl Opti-MEM I Reduced Serum Medium (31985062, Gibco) premixed with 3.75 μl Lipofectamine 3000 was pooled with 125 μl Opti-MEM Reduced Serum Medium containing 2.5 μg plasmid and 5 μl p3000 reagent. The solution was incubated for 15 min at room temperature (RT) and added dropwise to the medium on the cells (2 ml DMEM+/+ per well). The medium was replaced with fresh DMEM+/+ after 24 h. Cell culture experiments were performed 48 h post transfection. For transfection in 100 mm culture dishes, the reagent volumes were scaled up according to the surface growth area. The following plasmids were used for the transfection of COS7 cells: pcDNA4/HisMaxC (empty vector control); pcDNA4/HisMaxC-m*Pla2g4d*; pcDNA3.1(+)/myc-HisB-h*PLA2G4D*; and pcDNA4/HisMaxC-m*Pla2g4d*-S370A.

### Stable expression of PLA2G4D in COS7 cells

To produce lentiviral particles encoding mPLA2G4D, HEK293T cells (CRL-3216, ATCC) were seeded into 6-well plates (1 × 10^6^ cells/well). On the next day, cells were washed once with PBS and incubated for 5 h with DMEM (without FBS) containing 25 μM chloroquine (C6628, Sigma-Aldrich). Subsequently, cells were transfected according to the following protocol: 90 μl Opti-MEM I Reduced Serum Medium (Gibco) were premixed with the lentiviral packaging vectors psPAX2 (12260, Addgene) and pMD2.G (12,259, Addgene) as well as the pLVX-puro-6xHis-m*Pla2g4d* (1.6 μg of each plasmid) or the empty pLVX-puro vector as control. This mix was pooled with 90 μl Opti-MEM Reduced Serum Medium containing 30 μg polyethyleneimine (26292, Polysciences, Warrington). The solution was incubated for 20 min at RT and added dropwise into the medium on the cells. The next day, the medium was changed to DMEM +/+. On days 3 and 4 posttransfection, the cell culture medium harboring the lentiviral particles was collected, filtered through a 45 μm filter, and used for the transduction of COS7 cells. Therefore, 2 × 10^5^ cells/well were seeded into 6-well plates. On the following day, medium was changed to a mixture of 1 ml medium containing the lentiviral particles and 2 ml of DMEM +/+ supplemented with 8 μg/ml polybrene (H9268, Sigma-Aldrich). The 6-well plate was centrifuged for 1 h at 200 *g* (37°C), and cells were cultured overnight. Subsequently, transduced cells were selected by cultivation with DMEM +/+ supplemented with 1 μg/ml puromycin for several days (medium change every second day). Cells were expanded in the presence of puromycin (1 μg/ml), and protein expression was confirmed by Western blotting.

### Protein expression in Expi293F cells

For the purification of PLA2G4D, Expi293F cells were transfected in 10 ml or 25 ml culture volumes, respectively. Therefore, cells were seeded with a density of 2.5 × 10^6^ cells/ml in the respective culture flask (see Cell culture). On the following day, density was adjusted to 3 × 10^6^ cells/ml for transfection. A 25 ml culture was transfected according to the manufacturer's protocol: 1.5 ml Opti-MEM I Reduced Serum Medium containing 80 μl ExpiFectamine 293 Reagent (A14524, Gibco) was mixed with 1.4 ml Opti-MEM I containing 25 μg plasmid DNA. The mixture was incubated for 15 min at RT and added dropwise to the cell suspension. Eighteen to twenty-two hours posttransfection, 150 μl of ExpiFectamine 293 Transfection enhancer 1, and 1.5 ml of ExpiFectamine 293 transfection enhancer 2 (A14524, Gibco) were added to the culture. Respective volumes were scaled down for the transfection of 10 ml cultures. Forty-eight hours posttransfection cells were washed 3 times in 10 ml PBS and harvested for further experiments.

### Purification of PLA2G4D

Expi293F cells transfected with PLA2G4D encoding plasmids (pcDNA4/HisMaxC-m*Pla2g4d*; pcDNA3.1(+)/myc-HisB-h*PLA2G4D*; pcDNA4/HisMaxC-m*Pla2g4d*-S370A) were lysed in 60 mM sodium phosphate buffer (pH 7.4) containing 0.1% NP-40 and protease inhibitor (1 μg/ml pepstatin, 2 μg/ml antipain, and 20 μg/ml leupeptin) using the Sonopuls ultrasonic homogenizer (Bandelin, Berlin, Germany) equipped with a TT13 sonotrode (3 min with 0.5 s on/off cycle; amplitude 30%). The lysate was centrifuged at 1,000 *g* to remove cell debris and nuclei. The supernatant was adjusted to a concentration of 50 mM sodium phosphate and 300 mM NaCl and diluted to a volume of 50 ml with binding buffer (50 mM sodium phosphate buffer containing 300 mM NaCl) in a 50 ml tube. Five hundred microliters of the TALON metal affinity resin (635501, Takara Bio, Kusatsu, Japan) were equilibrated by resuspending the beads in 10 ml of binding buffer twice. Subsequently, the beads were mixed with the diluted lysate and the suspension was incubated overnight on an overtop shaker at 4°C. The TALON beads were removed from the lysate by centrifugation at 300 *g* for 3 min and transferred to a gravity column. The beads were washed 4× with 4 ml binding buffer. After that, the protein was eluted with 50 mM sodium phosphate buffer (pH 7) containing 300 mM NaCl and 250 mM imidazole in twenty 100 μl elution steps. To monitor protein elution, 5 μl of eluate per fraction was incubated with 200 μl of Protein Assay reagent (5000006, Bio-Rad, Hercules). The protein-containing fractions were pooled and desalted using the Econo-Pac® 10-DG Desalting Columns (732-2010, Bio-Rad). For this purpose, the column was equilibrated with 20 ml of storage buffer (50 mM Tris–HCl pH 7.4, 20 μM DTT, 250 mM sucrose) before loading the pooled fractions. Protein aliquots were frozen in liquid N_2_ and stored at −80°C.

### Site-directed mutagenesis

Mutagenesis was performed using the Q5 site-directed mutagenesis kit (E0552S, New England Biolabs, Frankfurt am Main, Germany) according to the manufacturer’s instructions using the following primers: forward: 5′-CAGTGGCATCgcgGGCTCTACAT-3′; reverse: 5′-AAGTAGGTCACACAGTCC-3′. This mutation exchanges the active site serine residue of mPLA2G4D with an alanine (S370A). Annealing temperatures were set according to the NCBI primer tool. Elongation time was set to 3:30 min (∼20–30 s/kb). Constructs were transformed into chemically competent *Escherichia coli*. The base-exchange was verified by Sanger sequencing.

### In vitro activity assays

Activity assays were performed by incubating 1 μg of partially purified proteins (2 μl for mPLA2G4D; 4 μl for hPLA2G4D; 10 μl for mPLA2G4D S370A) with 20 μl substrate solution containing 1 mM of the emulsified lipid supplemented with 2% FA-free BSA in PBS (pH 7.4) in the presence and absence of 1 mM CaCl_2_ for 1 h at 37°C. For substrate preparation, the organic phase of the lipid stock was dried under a stream of N_2_ and lipids were emulsified in substrate buffer using the Sonopuls ultrasonic homogenizer (Bandelin) equipped with a T72 sonotrode for 3 × 10 s with an amplitude of 15%. If not stated otherwise, the lipid substrates used were esterified with oleic acid (18:1) and are listed in supplemental Table S1. The reaction was stopped either by organic solvents for lipid extraction and TLC analysis or by the addition of glycerol reagent (F6428, Sigma-Aldrich, St. Louis) or NEFA-HR(2) enzymatic test (434-91795, 43-91995, FUJIFILM WAKO Chemicals, Neuss, Germany). Phospholipid:lyso-phospholipid-, monoacylglycerol:monoacylglycerol- (MMAT), phospholipid:monoacylglycerol- (PMAT), and phospholipid:diacylglycerol- (PDAT) transacylase assays were performed as described above using a substrate-containing equimolar concentrations (0.5 mM each) of acyl-group donor and acceptor in the absence of CaCl_2_. The indicated lipid substrates used in these assays are listed in supplemental Table S1. The reaction was stopped by the addition of an organic solvent mix used for lipid extraction and mass spectrometry analysis.

The in vitro diacylglycerol (DAG) and triacylglycerol (TAG) hydrolase activities were determined according to established protocols with minor adjustments (13). One microgram of partially purified protein in a total volume of 25 μl enzyme storage buffer (50 mM Tris–HCl pH 7.4, 20 μM DTT, 250 mM sucrose) was incubated with 25 μl substrate containing 0.32 mM 18:1 TAG (20,000 cpm/nmol TAG) and 45 μM phosphatidylcholine (PC)/phosphatidylinositol (PI) (3:1) or 0.3 mM *rac*-1,3 DAG (30,000 cpm/nmol DAG) and 50 μM PC/PI (3:1), respectively. Lipids were emulsified in 100 mM potassium phosphate buffer (pH 7.4) supplemented with 5% BSA (FA free). After 1 h at 37°C, the reaction was stopped by adding 650 μl of methanol/chloroform/heptane (v/v/v, 10/9/7) and 200 μl of 0.1 M potassium carbonate, 0.1 M boric acid (pH 10.5). After centrifugation at 1,000 *g* for 10 min, the radioactivity in 100 μl of the upper phase was determined by liquid scintillation using AquaLight Beta (461-035, Hidex, Turku, Finland) and a Tri-Carb 2900TR liquid scintillation analyzer (PerkinElmer, Waltham). Counts from control incubations containing enzyme storage buffer alone were subtracted and the rate of FA hydrolysis was calculated based on the specific radioactivity of the diolein or triolein substrate.

### Thin layer chromatography

Samples analyzed with TLC were extracted using a modified version of Matyash *et al.* (14). Lipids were extracted with 1 ml of methyl *tert*-butyl ether/methanol (v/v, 3/1) per sample, and phase separation was achieved by addition of 200 μl double distilled water (ddH_2_O). After centrifugation at 14,000 *g* for 5 min, 700 μl of the organic phase were transferred to a 2 ml tube and dried under a stream of N_2_. Samples were reconstituted in 30 μl chloroform and spotted onto a TLC Silica gel 60 aluminum sheet (1.05553.0001, Merck, Darmstadt, Germany). Chromatography of neutral lipids (MAG/DAG/TAG) was performed first with chloroform/acetone/acetic acid (v/v/v, 88/12/1; two-thirds of the plate) and a second time with toluol (full plate). Chromatography of polar lipids (phospholipids/lyso-phospholipids) was performed with chloroform/methanol/water (v/v/v, 65/35/5). Lipid bands were visualized via charring at 130°C after the silica plate was immersed in charring solution (25% ethanol v/v, 8.5% H_3_PO_4_ v/v, 5% w/v, CuSO_4_). Images were taken using the ChemiDoc™ Touch Imaging System (Bio-Rad), and densitometric analysis was done using the ImageJ software (15).

### SDS-PAGE and Western blotting

Protein samples were mixed with SDS-sample buffer (0.05 M Tris–HCl, 0.1 M DTT, SDS 2% w/v, 1.5 mM bromophenol blue, 1.075 M glycerol) and denatured at 95°C for 10 min. SDS-gel electrophoresis was performed in Tris-glycine buffer (20 mM Tris, 160 mM glycine, 0,083% SDS w/v) using a 10% polyacrylamide gel. The Color Prestained Protein Standard Broad Range (P7719S, New England Biolabs) was used as molecular weight reference. Proteins were transferred onto a PVDF membrane via the wet blotting method in CAPS buffer (10 mM CAPS, 10% v/v, methanol, pH 11). The membrane was blocked with 10% blotting grade milk powder diluted in Tris-buffered saline with Tween20 (20 mM Tris, 150 mM NaCl, 0.1% Tween 20, pH 7.4). For immunological detection, the primary antibodies in combination with antibody-host targeting secondary antibodies were used, and are listed in supplemental Table S2.

### RNA isolation and RT-PCR

RNA was isolated from cells or pulverized tissues using the TRIZol Reagent (15596026, Invitrogen) according to the manufacturer’s instructions. RNA was resuspended in 15–50 μl nuclease-free water. For cDNA synthesis, 1 μg of RNA was incubated with 1 unit DNaseI (M0303, New England Biolabs). After the addition of EDTA and heat inactivation, reverse transcription was performed using the LunaScript RT Supermix Kit (E3010, New England Biolabs) according to manufacturer’s instructions. cDNA was diluted 1:5 and RT-PCR was performed with the StepOnePlus Real-Time PCR System (4376600, Applied Biosystems, Waltham) using the SsoAdvanced Universal SYBR Green Supermix (1725274, Bio-Rad) together with gene targeting forward and reverse primers (10 pmol each). All primers are listed in supplemental Table S3.

### Immunocytochemistry

For immunocytochemistry, 5 × 10^3^ COS7 cells with stable PLA2G4D overexpression were seeded into 8-well culture chamber slides (94.6190.802, Sarstedt, Nümbrecht, Germany). After 24 h, cells were either treated with 200 μl DMEM +/+ (+1 μg/ml puromycin) containing 5 μM Thapsigargin (T9033, Sigma-Aldrich) and 5 μM calcimycin A23187 (C7522, Sigma-Aldrich) or DMSO as vehicle control for 15 min. Subsequently, cells were washed once with 200 μl PBS and fixed with 200 μl 4% paraformaldehyde for 15 min. Permeabilization was performed with 0.1% Triton X-100 in PBS for 10 min. Cells were washed 3 × 10 min with 200 μl PBS and blocked using 3% BSA in PBS for 1 h. After that, cells were incubated with an anti-6xHis tag antibody (ab18184, Abcam, Cambridge) diluted 1:200 in 3% BSA overnight at 4°C. The washing step was repeated (3 × 10 min with PBS) and the secondary anti-mouse Alexa Fluor® 594 Conjugate (8890, Cell Signaling Technology, Danvers) was added to the cells. Cells were washed again 3 × 10 min with PBS and subsequently covered with VECTASHIELD® Antifade Mounting Medium (H-1000, Vector Laboratories Inc, Newark) supplemented with 1 μg/ml DAPI.

Imaging data were acquired with the HC PL APO CS 63×/1.12 water immersion objective using the Leica SP8 LIGHTNING confocal microscope (Leica Microsystems, Wetzlar, Germany). A 405 nm diode laser (intensity at 2.8% of total output power) was used for the excitation of DAPI and its fluorescence emission was monitored with a photomultiplier tube between 417 nm and 462 nm. The Alexa Flour 594® antibody was excited with a 561 nm DPSS laser (intensity at 4.5% of total output power) and fluorescence emission was monitored with a photomultiplier tube between 614 nm and 648 nm. Image data were acquired in z-stacks with a resolution of 1,024 × 1,024 pixels with a scan speed of 600 Hz in unidirectional scan mode. Imaging data is presented either as maximum intensity projection of the z-stacks or as a single image plane and was visualized using different look-up tables. Imaging data were processed using the LasX and ImageJ software (15).

### Cell fractionation

For cell fractionation, four 100 mm dishes were seeded with 1.2 × 10^6^ COS7 cells, and cells were transfected with the pcDNA4/HisMaxC-m*Pla2g4d* expression vector. Forty-eight hours posttransfection, cells were washed once with PBS and harvested using a cell scraper. Cells were pooled and divided equally into four parts. Cell aliquots were centrifuged at 300 *g* for 3 min and resuspended in 1.1 ml 20 mM Tris/HCl buffer (supplemented with 250 mM sucrose, 1 μg/ml pepstatin, 2 μg/ml antipain, 20 μg/ml leupeptin, pH 7.4) either containing *i)* no CaCl_2_ and no EDTA, *ii)* no CaCl_2_ and 10 mM EDTA, *iii)* 2 mM CaCl_2_ and no EDTA, or *iv)* 2 mM CaCl_2_ and 10 mM EDTA. Mild cell lysis was performed by aspirating the suspension through a 26G needle 25 times. Cell lysates were centrifuged at 1,000 *g* for 10 min (4°C) to pellet the nuclei and cell debris. One hundred microliters of the 1,000 *g* supernatant were collected as the total fraction, and the residual 1 ml was centrifuged at 100,000 *g* for 60 min (4°C) to pellet the membrane fraction. The supernatant was collected (cytosolic fraction) and the pellet was washed three times with the respective buffers listed above, which were additionally supplemented with 2% Triton X-100. Subsequently, the pellet was emulsified in the respective buffer (containing 2% Triton X-100) via sonication. The protein content in each fraction was determined with Pierce BCA reagent (23225, Thermo Fisher Scientific) using BSA standard dilutions for each buffer condition. Five micrograms of protein was used for Western blotting analysis. Cell fractionation into heavy mitochondrial (3,000 *g* pellet), light mitochondrial (17,000 *g* pellet), microsomal (100,000 *g* pellet), and cytosolic fraction (100,000 *g* SN) was performed as described above by lysing mPLA2G4D transfected cells in the absence or presence of 2 mM CaCl_2_.

### Lipid analysis of COS7 cells

For targeted mass spectrometry analysis, COS7 cells were transfected with pcDNA4/HisMaxC-m*Pla2g4d* or pcDNA3.1(+)/myc-HisB-h*PLA2G4D* as well as the empty vector (pcDNA4/HisMaxC) or pcDNA4/HisMaxC-m*Pla2g4d*-S370A as control in 6-well plates. Forty-eight hours after transfection cells were washed twice with PBS and harvested using a cell scraper. Further details are provided in the mass spectrometry section.

For the MAG loading experiments, cells were preincubated with DGAT1 and DGAT2 inhibitors (16, 17) (PZ0207, PZ0233, Sigma-Aldrich, 5 μM each) or DMSO as vehicle control (1 ml of medium/well) for 30 min. Subsequently, the medium was changed to DMEM +/+ comprising inhibitors or DMSO as control but containing 300 μM *rac*-18:1 MAG (M7765, Sigma Aldrich) for 3.5 h. MAG was emulsified in cell culture medium in an ultrasonic bath (30 s) followed by sonication with a Sonopuls ultrasonic homogenizer for 3 × 10 s with an amplitude of 15%. After the incubation, cells were washed two times with 1 ml PBS, and cellular lipids were extracted directly from the 6-well plates using 1 ml hexane/isopropanol (v/v, 3:2) for 10 min, followed by 0.5 ml hexane/isopropanol (v/v, 3:2) for 5 min and 0.5 ml 100% isopropanol for 5 min. Extracts were pooled, dried under a stream of N_2_, and analyzed by TLC using chloroform/acetone/acetic acid (v/v/v, 88/12/1; two-thirds of the plate) as the first mobile phase and toluol as the second (full plate). Cell protein in the 6-well plates was dissolved in 400 μl NaOH/SDS (0.3 M/0.1% w/v) overnight and protein concentration was determined with Pierce BCA reagent (23225, Thermo Fisher Scientific) and used for normalization.

For experiments using radiolabeled lipids, COS7 cells transfected with pcDNA4/HisMaxC-m*Pla2g4d* or the empty vector control were loaded with 300 μM *rac*-18:1 MAG containing ^14^C-glycerol-backbone–labeled 2-18:1 MAG as tracer (1,500 cpm/nmol), or 300 μM ^3^H-labeled–oleic acid (3:1 BSA complex; 5,000 cpm/nmol) (ART0198; Hartmann Analytic, Braunschweig, Germany) for 4 h. The radioactive MAG was produced by digesting ^14^C-glycerol-backbone–labeled triolein (ARC3076; Hartmann Analytic) with a sn-1,3 specific lipase from *Candida antarctica* (L477, Sigma-Aldrich) and subsequent isolation of 2–18:1 MAG using Strata NH_2_ solid-phase extraction tubes (8B-S009-HBJ, Phenomenex, Aschaffenburg, Germany). After washing the cells 3 times in PBS, lipids were isolated with hexane/isopropanol as described above and spiked with nonradiolabeled MAG, DAG, and TAG to allow the visualization of TLC separated lipid bands in iodine-vapor. Phospholipid, MAG, DAG, and TAG bands were cut from the silica plate and analyzed via liquid scintillation using AquaLight Beta (461-035, Hidex, Turku, Finland) and a Hidex 600SL liquid scintillation analyzer. Data was normalized to cell protein.

### Animals

Mice were maintained on a regular light-dark cycle (14 h light, 10 h dark) at a RT of 23 ± 1°C and kept ad libitum on a standard laboratory chow diet (4.5% w/w fat, Ssniff Spezialdiaeten, R/M-H Extrudate, V1126-027). The CRISPR-Cas9 generated *Pla2g4d*-KO mice C57BL/6NJ-Pla2g4d^em1(IMPC)J^/Mmjax (MMRRC Strain: #051175-JAX) were obtained from the Jackson Laboratory (Bar Harbor, ME). Heterozygous animals were backcrossed four times on a C57BL/6NJ genetic background before switching to a homozygous breeding regime. All studies involving animals are reported in accordance with the ARRIVE guidelines for reporting experiments involving animals. Experimental procedures were approved by the Ethics committee of the University of Graz, and the Austrian Federal Ministry of Education, Science and Research (protocol number 2023-0.288.364) and were conducted in accordance with the council of Europe Convention (ETS 123). All animal procedures were performed as humanely as possible to minimize suffering.

### Isolation of primary keratinocytes from neonatal mice

Primary keratinocytes were isolated using a protocol adapted from Li *et al.* (18). In brief, two-day-old neonatal mice (WT or *Pla2g4d*-deficient) were sacrificed via decapitation and the skin was peeled off using forceps and scissors. The skin was rinsed in PBS and incubated for 15 h in 1.5 ml EpiLife growth medium (MEPICF500, Gibco), supplemented with 0.06 mM CaCl_2_, HKGS (S0015, Gibco) and Primocin (ant-pm-05, Invivogen, Toulouse, France), containing 4 mg/ml Dispase II (04942078001, Roche Diagnostics, Mannheim, Germany). Subsequently, the epidermis was separated from the dermis and placed on a 500 μl drop of TrypLE Express Enzyme (12604013, Gibco) for 20 min in a petri-dish with the basal side of the epidermis facing the droplet. One milliliter EpiLife growth medium was added to the petri-dish and epidermis was vigorously rubbed back and forth to release single cells. The medium was collected and the process was repeated 2 more times. The cell suspension was passed through a 100 μm filter, centrifuged for 5 min at 180 *g*, and the cell pellet was resuspended in 1 ml EpiLife growth medium containing above mentioned supplements. Cells were counted using a hemocytometer and seeded in gelatin (G1393, Sigma-Aldrich) coated 12-well plates with a density of 3 × 10^5^ cells per well. Cells were cultured for 4 days with a medium change every other day. Afterward, cells were simulated with 20 ng/ml IL17A (7955-IL) and 20 ng/ml TNFα (210-TA, both R&D Systems, Minneapolis) for another 24 h. For both RNA sequencing and lipidomic analysis, two wells of a 12-well plate containing cells from the same animal were pooled. Keratinocytes isolated from biological replicates (WT or *Pla2g4d*-deficient) were isolated, cultured, and analyzed individually.

### Mass spectrometry analysis

Cell pellets or samples from in vitro assay reactions were extracted using a modified version of Matyash *et al.* (14) in 1 ml methyl *tert*-butyl ether/methanol (3/1, v/v) containing 1 nmol butylated hydroxytoluene, 0.01% acetic acid, and internal standards (IS): 40 pmol TAG 51:0, 13 pmol rac-17:0/17:0 DAG, 26 pmol rac-17:0 MAG, 50 pmol 17:0/17:0 PC (all Larodan, Solna, Sweden), 133 pmol 17:0/17:0 PE, 30 pmol 17:0/17:0 phosphatidylserine (PS), 8 pmol 17:1 lyso-phosphatidylcholine (LPC), 30 pmol 17:1 lyso-phosphatidylethanolamine (LPE), 50 pmol 17:1 lyso-phosphatidylserine (LPS), 2.66 pmol d18:1/17:0 ceramide (CER), 10 pmol d18:1/17:0 sphingomyelin (SM) (all Avanti Polar Lipids, Alabaster, AL). Total lipid extraction was performed under constant shaking for 30 min at RT. After the addition of 200 μl ddH_2_O and further incubation for 10 min at RT, the samples were centrifuged at 14,000 rpm for 5 min at RT to establish phase separation. Seven hundred microliters of the upper organic phase were collected and dried under a stream of N_2_. Lipids were resolved in 200 μl (in vitro assay samples) or 100 μl (cell samples) 2-propanol/methanol/ddH_2_O (7/2.5/1, v/v/v). The extracted cell proteins were dried and solubilized in NaOH/SDS (0.3 M/0.1% w/v) at 65°C for 4 h and the protein content was determined using Pierce BCA reagent (23225, Thermo Fisher Scientific).

For both targeted and untargeted LC-MS analysis an Agilent 1290 Infinity II UHPLC (Agilent Technologies, Santa Clara) equipped with an AQUITY UPLC BEH C18 (2.1 × 150 mm 1.7 μm) column (186002353, Waters, Milford) was employed for the chromatographic separation. A gradient elution with eluents H_2_O (A) and 2-propanol (B), each supplemented with 10 mM ammonium acetate, 0.1% formic acid, and 8 μM phosphoric acid was used for the separation of the analytes. The gradient started with 50% B for 0.5 min, increased to 80% B within 8.5 min, then to 100% B over 13 min, held at 100% B for 2.5 min, and finally returned to 50% B to re-equilibrate the column. The flow rate was 0.15 ml/min and the column was kept at 50°C.

Targeted mass spectrometric analysis of lipids in positive mode was conducted using a 6470 Triple Quadrupole mass spectrometer (Agilent Technologies) equipped with an Agilent Dual AJS ESI source. Lipid species were analyzed by multiple reaction monitoring with the following parameters: TAG: [M+NH4]+ to neutral loss of targeted FA, collision energy (CE) 24, Fragmentor 179; DAG: [M+H]+-[H2O] to NL of targeted FA, CE 16, Fragmentor 110; MAG: [M+NH4]^+^ to [RCOO]^+^, CE 8, Fragmentor 116; PE/LPE: [M+H]^+^ to NL of PE headgroup, CE 20, Fragmentor 131 for PE, CE 16, Fragmentor 111 for LPE; PS/LPS: [M+H]^+^ to NL of PS head group, CE 20, Fragmentor 131 for PS, CE 20, Fragmentor 121 for LPS; PC: [M+H]^+^ to *m/z* 184, CE 30, Fragmentor 180; LPC: [M+H]+ to *m/z* 184, CE 28, Fragmentor 179. Data acquisition was performed using MassHunter Data Acquisition software (B.10, Agilent Technologies). Lipidomic data were processed with MassHunter Workstation Quantitative Analysis for QQQ (Version 10.0, Agilent Technologies). Data were normalized for recovery and extraction efficiency by calculating analyte/IS ratios (AU) and expressed as AU/μg protein.

Untargeted mass spectrometric analysis was conducted using an Agilent 6560 Ion Mobility LC/Q-TOF mass spectrometer in Q-TOF mode equipped with an Agilent Dual AJS ESI source. The scan range was set from 150 to 1,700 *m/z* in negative mode and from 120 to 1,700 *m/z* in positive mode. Auto-MS/MS mode was used for MS/MS analyses with three spectra per second, precursor absolute threshold of 200 counts, and fixed collision energy of 25 V. Quality control samples were analyzed using iterative acquisition mode to increase the number of MS/MS spectra for improved lipid annotation. Raw data were processed using MS-DIAL (version 5.1.221218) to identify lipids and generate a target list (19). This target list was then used with Lipid Data Analyzer (version 2.8.3_1) for peak picking and integration (20). Peak areas and lipid labels were manually curated. Data were normalized to IS and total protein content.

For statistical analysis, R (version 4.1.2) and RStudio (version 2024.04.2/764) were used with the *dplyr* package (https://CRAN.R-project.org/package=dplyr). Multivariate analysis was conducted using the *lipidr* workflow and figures were generated using the *ggplot2* package (21, 22). Normal data distribution was confirmed with the Shapiro–Wilk normality test and significance was assessed with unpaired two-tailed Student’s *t* test combined with correction for multiple comparisons using the false discovery rate (FDR).

### RNA sequencing

RNA from primary WT and *Pla2g4d*-deficient keratinocytes was isolated using the TRIzol reagent according to the manufacturer’s instructions. Bulk mRNA sequencing was conducted by Novogene (Munich, Germany) employing a strand-specific cDNA library (TruSeq) and the Illumina NovaSeq 6000 sequencing platform with a paired-end 150 bp sequencing strategy. Quality assessment was carried out using FastQC, and the sequencing reads were aligned to the mouse mm39 reference genome using the splice-aware aligner STAR (v 2.7.10b). Read quantitation at the gene level was performed using FeatureCounts (v2.0.6) and genecode annotation (vM31). Differential gene expression analysis between the groups was performed using the R-package *DESeq2* (23), and the resulting *P* values were adjusted for multiple testing using the FDR approach based on the Benjamini–Hochberg method. Genes with average expression across all samples >10, more than 1.5-fold change and FDR <0.1 were considered significantly differentially expressed. Volcano plots were generated using R-package *EnhancedVolcano*. Gene set enrichment analysis was conducted using the R-package *clusterProfiler* (24). The raw and processed sequencing data is available on the NCBI gene expression omnibus platform under the following accession number: GSE277039.

### Electronic artwork

Figures were created with *Prism* version 9.5.1 (*GraphPad*), *Incscape* version 1.3.2, *R-Studio* version 2024.04.2, *VolcanoseR* web application, and *Genesis* version 1.8.1 (25, 26).

### Quantification and statistical analysis

Data of experiments performed in triplicate or more are presented as mean ± SD. Assuming normally distributed data and equal SD between groups, differences were analyzed using unpaired two-tailed Student’s *t* test, multiple unpaired two-tailed Student’s *t* test with Bonferroni post-hoc analysis, or one-way ANOVA followed by correction for multiple comparison with Bonferroni posthoc analysis. Statistical analysis of untargeted LC-MS and RNA sequencing data is described in the corresponding materials and methods. Group differences were considered statistically significant at *P* < 0.05 and are shown as: ∗*P* < 0.05; ∗∗*P* < 0.01; and ∗∗∗*P* < 0.001. Details are found in the relevant figure legends. Statistical analysis was performed using *Prism* version 9.5.1 (*GraphPad*) and *R-Studio* version 2024.04.2.

## Results

### PLA2G4D acts as phospholipase with broad head group specificity

**Fig. 1:**
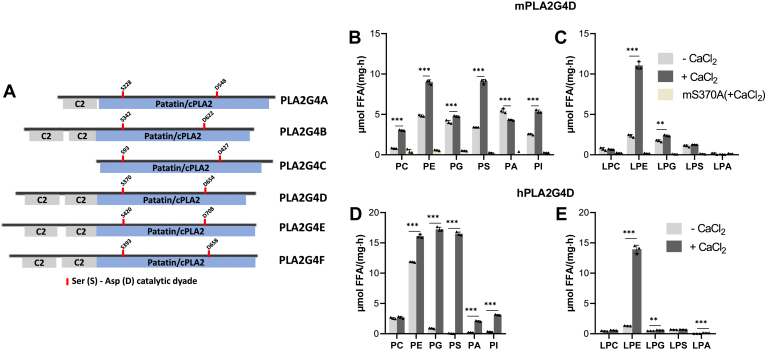
PLA2G4D acts as phospholipase with broad head group specificity. (A) Schematic illustration of the domain architecture of the mPLA2G4 family showing C2 domains (C2), the patatin-like domain (patatin/cPLA2), and the position of the serine (S) – aspartate (D) catalytic dyad. (B, C) (Lyso)phospholipase activity of partially purified murine PLA2G4D and the mS370A mutant in the absence and presence of 1 mM CaCl_2_. FFA release was determined using commercially available colorimetric kits. (D, E) (Lyso)phospholipase activity of partially purified human PLA2G4D in the absence and presence of 1 mM CaCl_2_. All enzyme activity assays were carried out by incubating 1 μg of partially purified protein with 20 μl of lipid substrate (1 mM) in PBS (pH 7.4) containing 2% BSA (FA free) for 1 h at 37°C (n = 3). Data are presented as mean ± SD. Statistical comparison was performed with multiple unpaired two-tailed Student’s *t* test, followed by Bonferroni posthoc analysis. Statistically significant differences are shown as: ∗*P* < 0.05; ∗∗*P* < 0.01; and ∗∗∗*P* < 0.001. PLA2G4, phospholipase A2 group IV.

The PLA2G4 family comprises six members that share structural similarities and are referred to as PLA2G4A-F or cPLA2α-ζ. This family is characterized by a catalytic domain structurally similar to the plant lipid hydrolase patatin (patatin-like domain). All members, except PLA2G4C, contain N-terminal Ca^2+^-binding C2 domains that regulate enzyme function (27, 28) (Fig. 1A). Published data suggest that all PLA2G4 members exhibit phospholipase activity (27). To further characterize PLA2G4D, we monitored its hydrolase activity using different lipid substrates. Therefore, the murine enzyme (mPLA2G4D), the human ortholog (hPLA2G4D), and a catalytically inactive mPLA2G4D carrying an active site S370A mutation (mS370A) were expressed in Expi293F cells and partially purified using 6xHIS affinity chromatography (supplemental Fig. S1A–C). mPLA2G4D exhibited phospholipase activity in the absence and presence of CaCl_2_ (Fig. 1B). The addition of Ca^2+^ increased the hydrolysis of PC, PE, PS, and PI, while the hydrolysis of phosphatidylglycerol (PG) and phosphatidic acid (PA) was less affected. The enzyme also hydrolyzed *sn*-1-acyl lyso-phospholipids, whereby Ca^2+^ strongly enhanced the degradation of LPE (Fig. 1C). These activities were not detected with the mS370A mutant, suggesting that the active site serine residue 370 is essential for the catalytic activity of PLA2G4D (Fig. 1B and C).

Human PLA2G4D showed a more pronounced head group specificity under basal conditions and was more sensitive to Ca^2+^ stimulation in comparison to mPLA2G4D. In the absence of CaCl_2_, hPLA2G4D exhibited highest activity against the zwitterionic phospholipid PE, followed by PC, while anionic phospholipids and lyso-phospholipids were barely degraded (Fig. 1D and E). The hydrolytic activity against anionic phospholipids PG, PS, PA, PI, and zwitterionic LPE was strongly increased in the presence of CaCl_2_ (Fig. 1D and E).

### PLA2G4D catalyzes the transfer of FAs between phospholipids

**Fig. 2:**
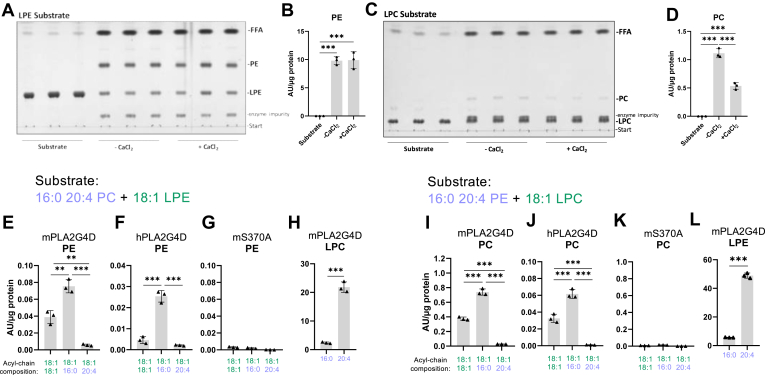
PLA2G4D catalyzes the transfer of fatty acids between phospholipids. (A, C) TLC analysis of PLA2G4D-derived reaction products using *sn-*1-18:1 LPE and *sn-*1-18:1 LPC as substrates (1 mM) in the absence and presence of 1 mM CaCl_2_. (B, D) Densitometric quantification of reaction products PE and PC is shown in A and C. (E–L) PLA2G4D-derived reaction products using an equimolar substrate mixture (0.5 mM each) of *sn-*1-16:0-*sn-*2-20:4 PC and *sn-*1-18:1 LPE, or *sn-*1-16:0-*sn-*2-20:4 PE and *sn-*1-18:1 LPC as donor and acceptor, respectively. The mS370A mutant was used as negative control (G, K). Lipids were analyzed by HPLC-MS. PE and PC subspecies are labeled according to their acyl-chain composition. All enzyme activity assays were carried out under conditions described in Figure 1 (n = 3). Data are presented as mean ± SD. Statistical comparison in (H, L) were performed with unpaired two-tailed Student’s *t* test, and in (B, D, E, F, I, J) with one-way ANOVA followed by Bonferroni posthoc analysis. Statistically significant differences are shown as: ∗*P* < 0.05; ∗∗*P* < 0.01; and ∗∗∗*P* < 0.001. LPC, lyso-phosphatidylcholine; LPE, lyso-phosphatidylethanolamine; PC, phosphatidylcholine; PE, phosphatidylethanolamine; PLA2G4, phospholipase A2 group IV.

We recently demonstrated that PLA2G4D is capable of catalyzing the acylation of the glycerol head group of PG and LPG, resulting in the formation of hemi-bis(monoacylglycero)phosphate and bis(monoacylglycero)phosphate, respectively (12). Using LPE or LPC as substrates, we also found transacylase activity generating PE or PC (Fig. 2A–D). Ca^2+^ did not affect the synthesis of PE (Fig. 2A and B), but inhibited PC formation by 50% (Fig. 2C and D). Both, mPLA2G4D and hPLA2G4D, catalyzed the FA transfer from PC to LPE (Fig. 2E and F) and from PE to LPC (Fig. 2I and J), while the S370A mutant showed no detectable activity (Fig. 2G and K). Using an equimolar substrate containing *sn-*1-16:0-*sn-*2-20:4 PC and *sn-*1-18:1 LPE as donor and acceptor, respectively, we observed the formation of PE containing 18:1-18:1, 18:1-16:0, and 18:1-20:4 acyl-chains in a ratio of 5.2/10/0.8 for mPLA2G4D and 1.8/10/0.8 for hPLA2G4D (Fig. 2E and F). Similar products were observed when the substrate contained *sn-*1-16:0-*sn-*2-20:4 PE and *sn-*1-18:1 LPC. Here, both orthologs generated 18:1-18:1, 18:1-16:0, and 18:1-20:4 PC in a ∼ 5/10/0.3 ratio (Fig. 2I and J). These observations suggest that PLA2G4D exhibits a preference for the *sn-*1-linked 16:0 acyl-chain of PE or PC. Accordingly, we identified 20:4 LPE and LPC as the most abundant deacylated reaction products (Fig. 2H and L).

### PLA2G4D acylates MAG and DAG

**Fig. 3:**
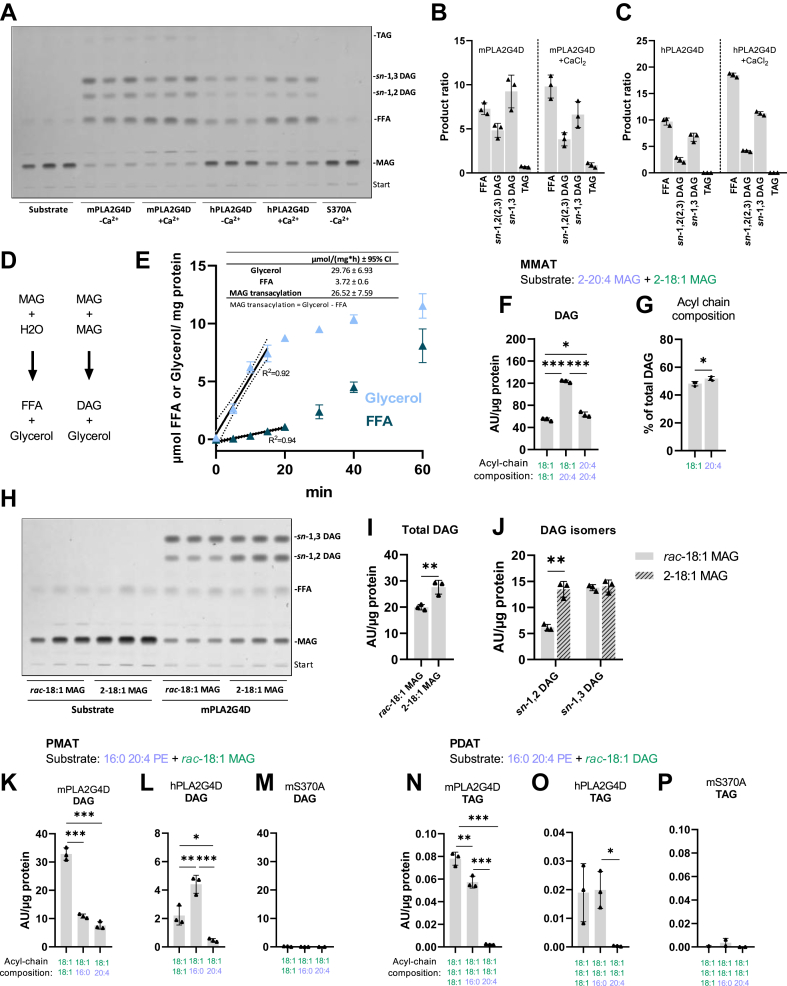
PLA2G4D acylates mono- and diacylglycerol. (A) TLC analysis of m- and hPLA2G4D-derived reaction products incubated with *rac*-18:1 MAG (1 mM) in the absence and presence of 1 mM CaCl_2_. The mS370A mutant was used as negative control. (B, C) Densitometric quantification of m- and hPLA2G4D-derived reaction products shown in (A). Data is presented as the relative ratio of products. (D) Schematic representation of MAG hydrolysis and transacylation reactions. (E) Time-dependent formation of glycerol and FFAs in the presence of mPLA2G4D using *rac*-18:1 MAG (1 mM) as substrate. The release of glycerol and FFAs was quantified using commercial kits. Linear regression was used to calculate the specific MMAT activity of the enzyme, based on the difference between glycerol and FFA release (insert). The dotted line indicates the 95% confidence interval (CI), and *R*^2^ shows the coefficient of determination. (F) mPLA2G4D-derived reaction products using an equimolar substrate mixture of 2–18:1 MAG and 2-20:4 MAG (0.5 mM each). Assay was performed in the absence of 1 mM CaCl_2_, and lipids were analyzed by HPLC-MS. DAG subspecies are labeled according to their acyl-chain composition. (G) Relative abundance of 18:1 and 20:4 acyl chains in total DAG, calculated from data shown in (F). (H) TLC analysis of mPLA2G4D-derived reaction products incubated with *rac-*18:1 MAG or 2-18:1 MAG (0.5 mM each) in the absence of CaCl_2_. (I) Total DAG levels calculated as sum of *sn*-1,2 and *sn*-1,3-DAG band intensities shown in (H). (J) Abundance of *sn*-1,2 and *sn*-1,3-DAG isomers, based on band intensities shown in (H). (K–P) PLA2G4D-derived reaction products using an equimolar substrate mixture (0.5 mM each) of *rac*-18:1 MAG and *sn-*1-16:0-*sn-*2-20:4 PE, or *rac*-18:1 DAG and *sn-*1-16:0-*sn-*2-20:4 PE, respectively. Assays were performed in the absence of CaCl_2_ and lipids were analyzed by HPLC-MS. All enzyme activity assays were carried out under conditions described in Figure 1 (n = 3). Data are presented as mean ± SD. Statistical comparisons in (F, K, L, N, O) were performed with one-way ANOVA followed by Bonferroni posthoc analysis and in (G, I, J) with unpaired two-tailed Student’s *t* test. Statistically significant differences are shown as: ∗*P* < 0.05; ∗∗*P* < 0.01; and ∗∗∗*P* < 0.001. DAG, diacylglycerol; MAG, monoacylglycerol; PE, phosphatidylethanolamine; PLA2G4, phospholipase A2 group IV; TAG, triacylglycerol.

Further characterization of the enzymatic activities of PLA2G4D revealed that the enzyme also uses acylglycerols as a substrate for transacylation reactions. When mPLA2G4D was incubated with *rac-*18:1 MAG, the reaction generated FFA, *sn-*1,2(2,3) DAG, *sn-*1,3 DAG, and TAG in a ratio of 8/5/10/1 (Fig. 3A and B). The addition of Ca^2+^ slightly increased hydrolysis and reduced DAG formation. Incubation of hPLA2G4D with *rac*-18:1 MAG resulted in the formation of FFA, *sn-*1,2(2,3) DAG, and *sn-*1,3 DAG with a ratio of 10/3/7, while TAG was not detected (Fig. 3A and C). Ca^2+^ slightly enhanced both the hydrolase and transacylase activity of hPLA2G4D. These observations suggest that PLA2G4D exhibits MAG:MAG and, at a lower rate, MAG:DAG or DAG:DAG acyltransferase activity (MMAT, MDAT, and DDAT activity, respectively).

Next, we compared hydrolase and transacylase activity of PLA2G4D in time-course experiments using *rac*-18:1 MAG as substrate. MAG hydrolysis generates equimolar amounts of glycerol and FFAs, while the transacylation reaction delivers glycerol and acylglycerols, but no FFAs (Fig. 3D). Hence, the difference between glycerol and FFA release corresponds to the rate of MAG transacylation, while FFA release indicates substrate hydrolysis. Activities were calculated in the linear range of glycerol and FFA release, which was within the initial 15–20 min of the reaction (Fig. 3E). PLA2G4D catalyzed MAG transacylation with a specific activity of 26.5 μmol/(mg∙h), while MAG hydrolysis was catalyzed at a rate of 3.7 μmol/(mg∙h) (Insert Fig. 3E), suggesting that the transacylation reaction occurs at a ∼ 7-fold faster rate than MAG hydrolysis. To compare transacylase and hydrolase activity, we determined time-dependent hydrolysis of the preferred phospholipid substrate PE in time-course experiments revealing a specific activity of 17.2 μmol/(mg∙h) (supplemental Fig. S2A). Thus, PE hydrolysis and MAG acylation reactions occur at comparable rates. Dose-response experiments showed that both reactions increased linearly up to 1 μg protein/assay (supplemental Fig. S2B and C). We also determined hydrolase activity against radiolabeled DAG or TAG emulsified in phospholipid micelles using established protocols (13). mPLA2G4D hydrolyzed DAG at a rate of 1.5 μmol/(mg∙h) independent of the presence of CaCl_2_. The enzyme showed low but detectable TAG hydrolase activity of 0.3 μmol/(mg∙h), which increased ∼ 4-fold upon CaCl_2_ addition (supplemental Fig. S2D and E). Similarly, hPLA2G4D showed DAG and TAG hydrolase activity, both stimulated by CaCl_2_ (supplemental Fig. S2D and E). These assays suggest that DAG and TAG hydrolysis occurs at substantial lower rates compared to phospholipase and transacylase reactions.

To analyze FA preferences of PLA2G4D, we monitored DAG formation in the presence of a substrate containing equimolar concentrations of 2–18:1 MAG and 2-20:4 MAG using LC-MS. The enzyme generated di-oleoyl- (18:1-18:1), oleoyl-arachidonoyl (18:1-20:4), and di-arachidonoyl (20:4-20:4) glycerol at a ratio of 1/2.3/1.2 (Fig. 3F). Analysis of total DAG-FA composition revealed approximately equal arachidonic acid and oleic acid incorporation. (Fig. 3G). To get insights into the positional preferences of PLA2G4D, we compared DAG formation using *rac*-18:1 MAG or 2-18:1 MAG as substrate (Fig. 3H–J). Total DAG levels were 1.4-fold higher using 2-18:1 MAG as substrate (Fig. 3H and I), due to increased *sn*-1,2 DAG formation (Fig. 3J), indicating a slight preference for or 2-18:1 MAG. The high level of *sn*-1,3 DAG using 2-18:1 MAG as substrate is likely to spontaneous acyl chain migration to *sn*-1,3 positions under the applied conditions (29).

Next, we investigated whether PLA2G4D exhibits phospholipid:MAG(PMAT) and PDAT activities. In these assays, we used *sn-*1-16:0-*sn-*2-20:4 PE as acyl-donor and *rac*-18:1 MAG or *rac*-18:1 DAG as acceptor. With *rac*-18:1 MAG as acceptor, m- and hPLA2G4D generated 18:1-18:1, 18:1-16:0, and 18:1-20:4 DAG at a ratio of 10/3.3/2.3 and 5/10/1, respectively (Fig. 3K and L), confirming that PLA2G4D has PMAT activity. Higher levels of 18:1-18:1 DAG generated by mPLA2G4D suggest a preference for the MMAT reaction, however, the sum of 18:1-16:0 and 18:1-20:4 DAG account for 56% of 18:1-18:1 DAG highlighting that both reactions occur at comparable rates (Fig. 3K). For hPLA2G4D, 18:1-16:0 DAG was the most abundant reaction product indicating a preference for the PMAT reaction (Fig. 3L). With *rac*-18:1 DAG as acceptor, the major products for m- and hPLA2G4D were 18:1-18:1-18:1 and 18:1-18:1-16:0 TAG (Fig. 3N and O). 18:1-18:1-20:4 TAG was produced at a much lower rate, indicating the preferential transfer of *sn-*1–esterified FA of phospholipids to DAG under the applied conditions. The mS370A mutant was used as negative control and showed no detectable activity (Fig. M and P).

### PLA2G4D is a cytosolic enzyme that interacts with membranes in a calcium-dependent manner

**Fig. 4:**
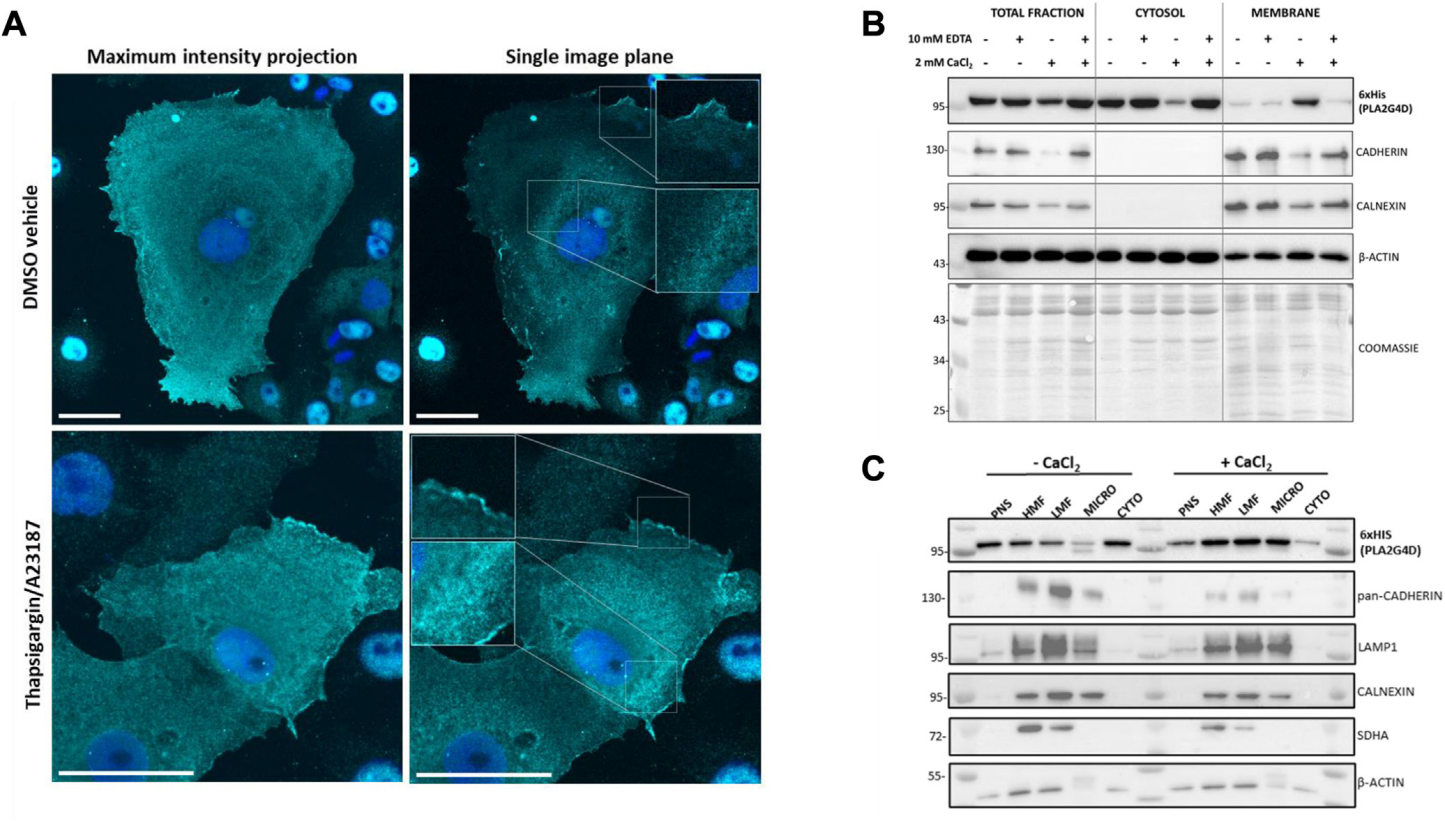
PLA2G4D is a cytosolic enzyme that interacts with membranes in a calcium-dependent manner. (A) Confocal imaging of COS7 cells stably overexpressing His-tagged mPLA2G4D. Cells were fixed with paraformaldehyde and stained with an anti-6xHis antibody (cyan). To increase cytosolic Ca^2+^ concentrations, cells were treated with thapsigargin/calcimycin A23187 (5 μM each) or DMSO for 15 min before fixation. Maximum intensity projection shows the highest pixel intensities from stacked images indicating that the enzyme shows primarily cytosolic localization. Zoomed sections from a single image plane suggest that PLA2G4D partially colocalizes with cytoplasmic and plasma membranes. Nuclei were stained with DAPI (blue). Scale bar: 40 μm. Data are representative for two independent experiments. (B) Western blotting analysis of total (1,000 *g* supernatant), cytosolic (100,000 *g* supernatant), and membrane fractions (100,000 *g* pellet) of COS7 cells overexpressing His-tagged mPLA2G4D. Cell fractionation was performed in the absence and presence of 2 mM CaCl_2_ and 10 mM EDTA. The plasma membrane protein Cadherin and ER protein calnexin were used as markers for the membrane fractions. β-Actin and Coomassie staining served as loading control. Data are representative for two independent experiments. (C) Western blotting analysis of total fraction (TF; 1,000 *g* supernatant), heavy mitochondrial fraction (HMF; 3,000 *g* pellet), light mitochondrial fraction (LMF; 17,000 *g* pellet), microsomal fraction (MICRO; 100,000 *g* pellet), and cytosolic fraction (CYTO; 100,000 *g* supernatant) of COS7 cells overexpressing His-tagged mPLA2G4D. Cell fractionation was performed in the absence and presence of 2 mM CaCl_2_. Lamp1, Calnexin, pan-Cadherin, and SDHA were used as marker-proteins for the endo-lysosomal compartment, ER, plasma membrane, and mitochondria, respectively. β-Actin served as loading control. PLA2G4, phospholipase A2 group IV.

To study the subcellular localization of PLA2G4D, we conducted immunocytochemistry with COS7 cells stably expressing the His-tagged mPLA2G4D. The majority of the protein was present in the cytosol, but we also observed localization on the plasma membrane and cytoplasmic membrane structures (Fig. 4A). Treatment of cells with thapsigargin and calcimycin (A23187), which increases cytosolic Ca^2+^ concentrations, did not induce major changes in subcellular localization (Fig. 4A). To further explore the role of Ca^2+^ in regulating membrane interaction, we investigated the association of PLA2G4D with cytosolic and membrane fractions in the absence and presence of CaCl_2_. When CaCl_2_ was absent during cell fractionation and in the presence of the Ca^2+^-chelator EDTA, most of PLA2G4D was found in the cytosolic fraction (Fig. 4B). Conversely, in the presence of CaCl_2_, the enzyme was detected primarily in the membrane fraction, and membrane binding was prevented using an excess of EDTA (Fig. 4B). Next, we performed a crude separation of membrane fractions by sequential centrifugation revealing heavy mitochondrial fractions (HMFs, 3,000 *g* pellet), light mitochondrial fractions (LMFs, 17,000 *g* pellet), and microsomal fractions (100,000 *g* pellet). Again, we observed a prominent PLA2G4D band in the cytosolic fraction and little signal in the microsomal fraction in the absence of CaCl_2_ (Fig. 4C). The enzyme was also detected in both the HMF and LMF, which were enriched in marker proteins for mitochondria (SDHA), endo-lysosomes (LAMP1), as well as endoplasmic reticulum and plasma membrane (Calnexin, Cadherin). In the presence of CaCl_2_, we observed a strong increase in PLA2G4D signal in the microsomal fraction, a moderate increase in the HMF and LMF, and a strong decrease in the cytosolic fraction (Fig. 4C). These data indicate that PLA2G4D interacts with membranes from multiple organelles in a Ca^2+^-dependent manner.

### PLA2G4D overexpression alters cellular acylglycerol and phospholipid profiles

To obtain first insights into the cellular function of the enzyme, we performed gain-of-function studies in COS7 cells, which lack endogenous *Pla2g4d* mRNA expression (supplemental Fig. S6). Experiments were performed with constructs encoding for active proteins or the catalytically dead mS370A mutant as a control. Protein expression was confirmed by Western blotting analysis (supplemental Fig. S3A).

**Fig. 5:**
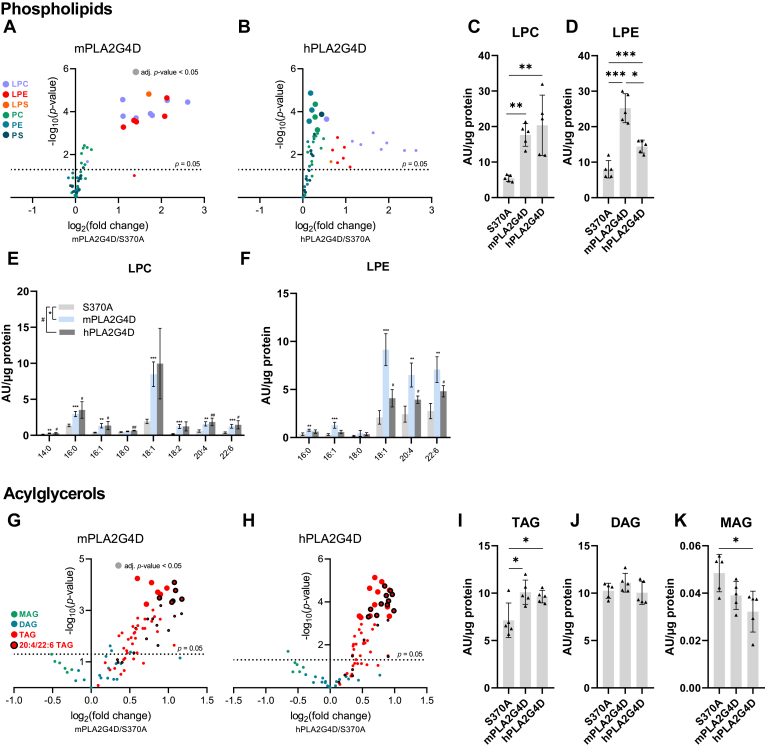
PLA2G4D overexpression alters cellular acylglycerol and phospholipid profiles. (A, B) Volcano plots showing changes in glycerophospholipids in m- and hPLA2G4D-expressing COS7 cells compared to control cells expressing the inactive mS370A mutant. The *x*-axis shows the log_2_ (fold change) of the indicated phospholipid subspecies. The *y*-axis shows the -log_10_(*P* value). Lipids were analyzed by HPLC-MS 48 h posttransfection. (C, D) Total LPC and LPE levels calculated based on the sum of signals from all detected subspecies. (E, F) Lipid subspecies of LPC and LPE in COS7 cells expressing m-, hPLA2G4D, or the mS370A mutant. (G, H) Volcano plots showing changes of MAG, DAG, and TAG subspecies in m- and hPLA2G4D-expressing cells compared to control cells expressing the mS370A mutant. (I–K) Total MAG, DAG, and TAG levels calculated based on the sum of signals from all detected subspecies. Data are shown as mean ± SD (n = 5) and are representative of three independent experiments. Statistical comparison in (A, B, E, F, G, H) was performed with multiple unpaired two-tailed Student’s *t* test followed by Bonferroni posthoc analysis. Large-sized spots in volcano plots represent lipids with an adjusted *P* value < 0.05. Statistical comparisons in (C, D, I, J, K) were performed with one-way ANOVA followed by Bonferroni posthoc analysis. Statistically significant differences are shown as: ∗, #*P* < 0.05; ∗∗, ###*P* < 0.01; and ∗∗∗, ###*P* < 0.001. DAG, diacylglycerol; LPC, lyso-phosphatidylcholine; LPE, lyso-phosphatidylethanolamine; MAG, monoacylglycerol; PLA2G4, phospholipase A2 group IV; TAG, triacylglycerol.

Targeted lipidomic analysis of glycerophospholipids revealed that PLA2G4D-expressing cells accumulate saturated and unsaturated LPC and LPE subspecies (Fig. 5A and B), as shown in detail in Figure 5E and F. Total LPC and LPE levels were strongly increased in both m- and hPLA2G4D-expressing cells (Fig. 5C and D). Specifically, in hPLA2G4D-overexpressing cells, we observed slightly increased total PE levels (supplemental Fig. S3B), due to a moderate increase of several PE subspecies (Fig. S4H). Total PC and PS remained unchanged (supplemental Fig. S3C and D), although some subspecies were slightly elevated (supplemental Fig. S4I and J).

Overexpression of mPLA2G4D or hPLA2G4D caused a significant increase in several TAG subspecies (Fig. 5G and H) resulting in increased total TAG levels (Fig. 5I). The strongest effect was observed for TAG subspecies containing PUFAs such as arachidonic acid (20:4) and docosahexaenoic acid (22:6) (supplemental Fig. S4F, G, M and N). DAG levels and composition remained unaffected (Fig. 5J and S4E and L). Total MAG was reduced by 34% in hPLA2G4D-expressing cells, (Fig. 5K), due to a decrease in all analyzed subspecies (supplemental Fig. S4K).

### PLA2G4D enhances the incorporation of MAG from extracellular sources into cellular acylglycerols independently of DGAT

To study the role of PLA2G4D in the metabolization of MAG from extracellular sources, COS7 cells were supplemented with *rac*-18:1 MAG. Subsequently, we analyzed the effect of PLA2G4D overexpression on cellular acylglycerol accumulation by TLC. The experiment was conducted in the absence and presence of small molecule inhibitors targeting DGAT1 and DGAT2, blocking acyl-CoA–dependent TAG synthesis.

**Fig. 6:**
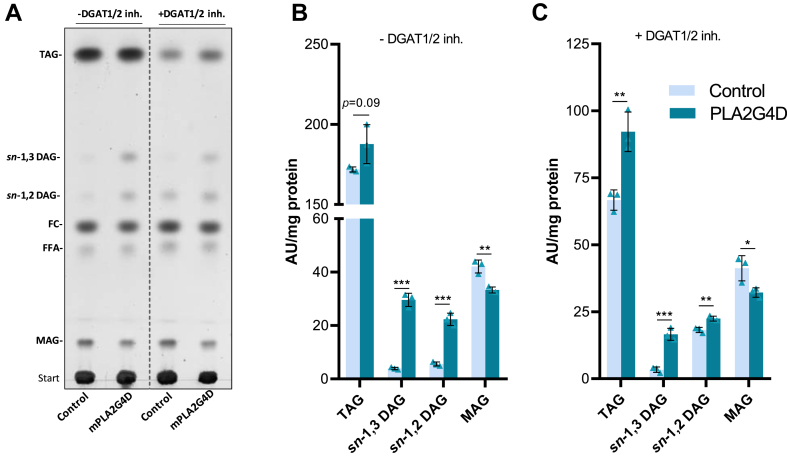
PLA2G4D enhances the incorporation of MAG from extracellular sources into cellular acylglycerols independently of DGAT. (A) Representative TLC of acylglycerol levels of control (empty vector transfection) and PLA2G4D-expressing COS7 cells loaded with 300 μM *rac*-18:1 MAG for 210 min in the absence and presence of DGAT1 and DGAT2 inhibitors (5 μM each). (B, C) Densitometric quantification of acylglycerol bands in *rac*-18:1 MAG loaded cells in the absence and presence of DGAT inhibitors (shown in A). Experiments were performed in triplicates (n = 3) and data are presented as mean ± SD and are representative for two independent experiments. Statistical comparison in all graphs was performed with an unpaired two-tailed Student’s *t* test. Statistically significant differences are shown as: ∗*P* < 0.05; ∗∗*P* < 0.01; ∗∗∗*P* < 0.001. DGAT, diacylglycerol acyltransferasel; FC, free cholestero; MAG, monoacylglycerol; PLA2G4, phospholipase A2 group IV.

In the absence of DGAT inhibitors, PLA2G4D-expressing cells showed a trend toward increased TAG levels (Fig. 6A and B). However, the most prominent effect was observed for DAG, which increased 5.5-fold due to a several-fold increase in both *sn*-1,2(2,3) and *sn*-1,3 DAGs (Fig. 6A and B). MAG levels were reduced by 22% (Fig. 6B). Changes in DAG and MAG are consistent with the high MMAT activity of PLA2G4D. Data were confirmed using ^14^C-backbone labeled 2-18:1 MAG (supplemental Fig. S5A). PLA2G4D-expressing cells showed a 3.7- and 5.4-fold increased incorporation of radioactivity into *sn*-1,2(2,3) and *sn*-1,3 DAGs, respectively, compared to control cells. The radioactivity in cellular MAGs was reduced by 70%. No significant changes were observed for polar lipids and TAG (supplemental Fig. S5A). Using ^3^H-labeled oleic acid as tracer, PLA2G4D expression did not affect the incorporation of radioactivity into polar and neutral lipids, presumably because FFAs are metabolized via acyl-CoA–dependent pathways (supplemental Fig. S5B).

Inhibition of DGAT activity decreased cellular TAG stores in both control and PLA2G4D-expressing cells, and increased DAG levels due to accumulation of *sn*-1,2(2,3) DAG (Fig. 6A). Under these conditions, PLA2G4D overexpression increased TAG accumulation 1.4-fold (Fig. 6C). Total DAG increased 1.8-fold, mainly due to accumulation of *sn*-1,3 DAG, while MAGs were again reduced by 22% (Fig. 6C). Thus, PLA2G4D overexpression increases TAG formation in the absence of DGAT activity. TAG synthesis may be promoted by PLA2G4D-catalyzed MDAT, DDAT, or PDAT reactions. Alternatively, the MMAT activity provides DAGs, which are further acylated by PLA2G4D- and DGAT-independent mechanisms.

### PLA2G4D is expressed in keratinocytes

**Fig. 7:**
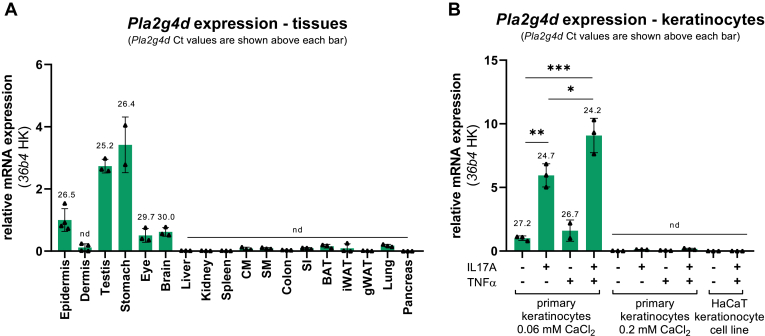
PLA2G4D mRNA expression in mouse tissues and keratinocytes. (A) *Pla2g4d* mRNA expression normalized to *36b4* housekeeping (HK) gene in murine tissues. Ct values for *Pla2g4d* are shown above each bar. Tissues were analyzed in 2–4 biological replicates. (B) *Pla2g4d* mRNA expression normalized to *36b4* HK gene in primary keratinocytes and the human HaCaT keratinocyte cell line (n = 3). Primary cells were isolated from the epidermis of two-day-old neonatal mice and cultured for four days in the presence of low CaCl_2_ concentrations (0.06 mM). Keratinocyte differentiation was induced by increasing CaCl_2_ to 0.2 mM for 24 h. Before RNA isolation, primary and HaCaT keratinocytes were cultured in absence and the presence of IL17A and/or TNFα (20 ng/ml each) for 24 h. Ct values above 30 were considered as no or negligible expression for *Pla2g4d* (nd). Data are shown as mean ± SD. Statistical comparisons in (B) were performed with one-way ANOVA followed by Bonferroni posthoc analysis. Statistically significant differences are shown as: ∗*P* < 0.05; ∗∗*P* < 0.01; and ∗∗∗*P* < 0.001. BAT, brown adipose tissue; CM, cardiac muscle; iWAT, inguinal white adipose tissue; gWAT, gonadal white adipose tissue; SM, skeletal muscle; SI, small intestine; PLA2G4, phospholipase A2 group IV.

Previous observations suggested that PLA2G4D is expressed in several tissues, including skin, testis, placenta, stomach, small intestine, and brain (30, 31). Accordingly, we detected *Pla2g4d* gene expression in murine testis, stomach, and epidermis as well as lower levels in the eye and brain (Fig. 7A). In line with data from public databases, we also found that PLA2G4D mRNA is poorly or not expressed in frequently used mammalian cell lines of different origin including COS7 cells (Ct values > 30) (supplemental Fig. S6). To find a cell system with high endogenous expression, we determined *Pla2g4d* mRNA levels in primary keratinocytes obtained from WT neonatal mice. In accordance with published data, we found that these cells show *Pla2g4d* expression (32) (Fig. 7B). *Pla2g4d* mRNA levels further increased in response to treatment with the cytokines TNFα and IL17A, while Ca^2+^-induced differentiation strongly reduced expression (Fig. 7B). No *PLA2G4D* mRNA was detected in the human HaCaT keratinocyte cell line in the absence and presence of cytokines (Fig. 7B).

### *Pla2g4d*-deficient keratinocytes show complex lipidomic changes

To study PLA2G4D function in a physiologically relevant system, we isolated keratinocytes from *Pla2g4d*-deficient mice and WT controls. The lack of *Pla2g4d* exon 3 mRNA was confirmed by RT-PCR and RNA sequencing (supplemental Fig. S7). *Pla2g4d*-KO mice are fertile, show normal growth and weight, and have no obvious skin or other phenotypes (not shown).

**Fig. 8:**
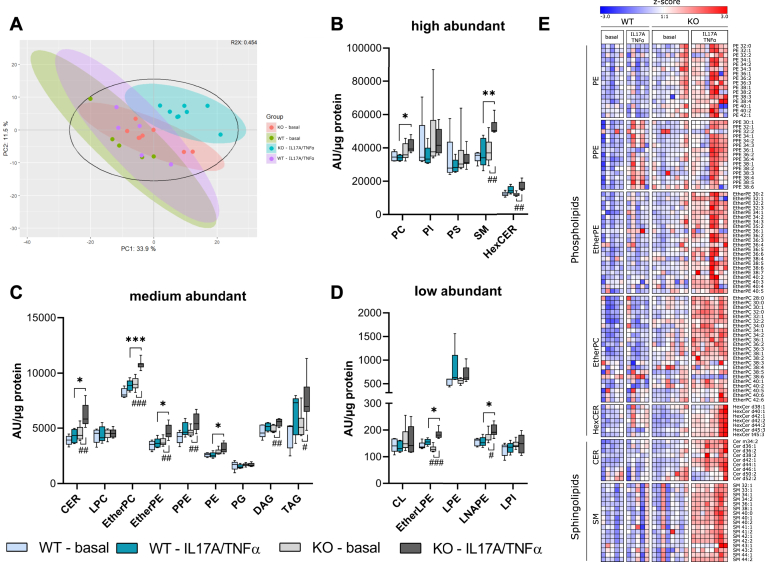
*Pla2g4d*-deficient keratinocytes show complex lipidomic changes. (A) Principal component analysis of untargeted HPLC-MS lipidomics data from primary keratinocytes isolated from two-day-old WT (n = 5) and *Pla2g4d*-deficient (KO; n = 8) neonatal mice. Cells were cultured for four days under low CaCl_2_ concentrations (0.06 mM) and subsequently stimulated with IL17A and TNFα (20 ng/ml each) for 24 h. (B–D) Total lipid class levels of basal and IL17A/TNFα treated WT and KO keratinocytes. Changes in lipid classes were calculated based on the sum of all analyzed subspecies. Based on signal intensities, classes are grouped in high, medium, and low abundance. (E) Heat maps displaying the z-score of PE, PPE, EtherPE, EtherPC, HexCER, CER, and SM subspecies were labeled by the number of C-atoms in fatty acids and the total number of double bonds. The z-score was calculated as number of SDs above or below from the mean of the dataset. Statistical comparisons were performed with multiple unpaired two-tailed Student’s *t* test with correction for multiple comparisons using the false discovery rate (FDR). Statistically significant differences are shown as: ∗, #*P* < 0.05; ∗∗, ##*P* < 0.01; and ∗∗∗, ###*P* < 0.001. CER, ceramide; CL, cardiolipin; LNAPE, lyso-N-acyl-PE; HexCER, hexosylceramide; PE, phosphatidylethanolamine; PLA2G4, phospholipase A2 group IV; PPE, plasmalogen-PE; SM, sphingomyelin.

Lipidomic analysis of cultured primary keratinocytes was performed under basal conditions and in the presence of IL17A and TNFα. Using untargeted HPLC-MS, we analyzed 296 different lipid species. Principal component analysis indicated the highest separation of cytokine-stimulated KO cells from other groups (Fig. 8A).

Changes in specific lipid classes are shown in Figure 8B–D. Under basal conditions, we did not find major differences between genotypes. Conversely, cytokine-treated KO cells showed significantly increased levels of several glycerophospholipid and sphingolipid species, including PE (1.2-fold), PC (1.2-fold), ether-PE (1.3-fold), ether-LPE (1.2-fold), lyso-N-acyl-PE (1.3-fold), ether-PC (1.2-fold), CER (1.5-fold), and SM (1.4-fold), when compared to cytokine-treated WT cells (Fig. 8B–D). The elevated levels of glycerophospholipids and sphingolipids were attributable to an increase in the majority of the molecular species identified (Fig. 8E). Total levels of PI, hexosylceramides, PS, LPC, plasmalogen-PE, PG, DAG, TAG, cardiolipin, and lyso-PI remained unaltered between genotypes under both basal and inflammatory conditions (Fig. 8B–D).

The most regulated molecular lipid species between genotypes are displayed in volcano plots (supplemental Fig. S8A and B). We did not observe any significant changes between genotypes under basal conditions (supplemental Fig. S8A). In the presence of cytokines, 23 species were elevated in KO compared to WT cells, while only ether-PC 38:6 was decreased (fold decrease or increase >1.5, adj. *P* value > 0.05) (supplemental Fig. S8B and C). Although total DAG and TAG levels were unchanged, seven TAG and two DAG subspecies were among the top 15 most regulated hits. All other upregulated species were glycerophospholipids and sphingolipids (supplemental Fig. S8B and C).

### *Pla2g4d*-deficient keratinocytes show complex transcriptional changes

PLA2G4D is expressed in keratinocytes and upregulated in psoriasis indicating a multifaceted role in the regulation of cell function (30, 32). To study its impact on gene expression in primary keratinocytes, we performed RNA sequencing analysis of KO and WT cells. The treatment of keratinocytes with psoriasis-promoting cytokines IL17A/TNFα strongly changed the transcriptome. In WT and KO cells, we identified 2,396 (1,488 up/908 down) and 1,855 (1,079 up/776 down) differentially expressed genes upon cytokine treatment, respectively (supplemental Fig. S9A, B, and E). In both genotypes, several psoriasis-related genes (*S100a8*, *S100a9*, *Sprr2e*, *Sprr2f*, *Lce3a*, *Lce3b*) were strongly upregulated, indicating that the cytokine stimulation induces a psoriasis-like phenotype (supplemental Fig. S9A and B). Comparison of genotypes under basal and inflammatory conditions revealed 509 (297 up/212 down) and 155 (32 up/122 down) differentially expressed genes, respectively (supplemental Fig. S9C–E). Principal component analysis of RNA sequencing data from all four groups revealed a clear segregation along PC1, primarily attributed to cytokine treatment, and a moderate separation along PC2, influenced by the genotypes (supplemental Fig. S9F).

**Fig. 9:**
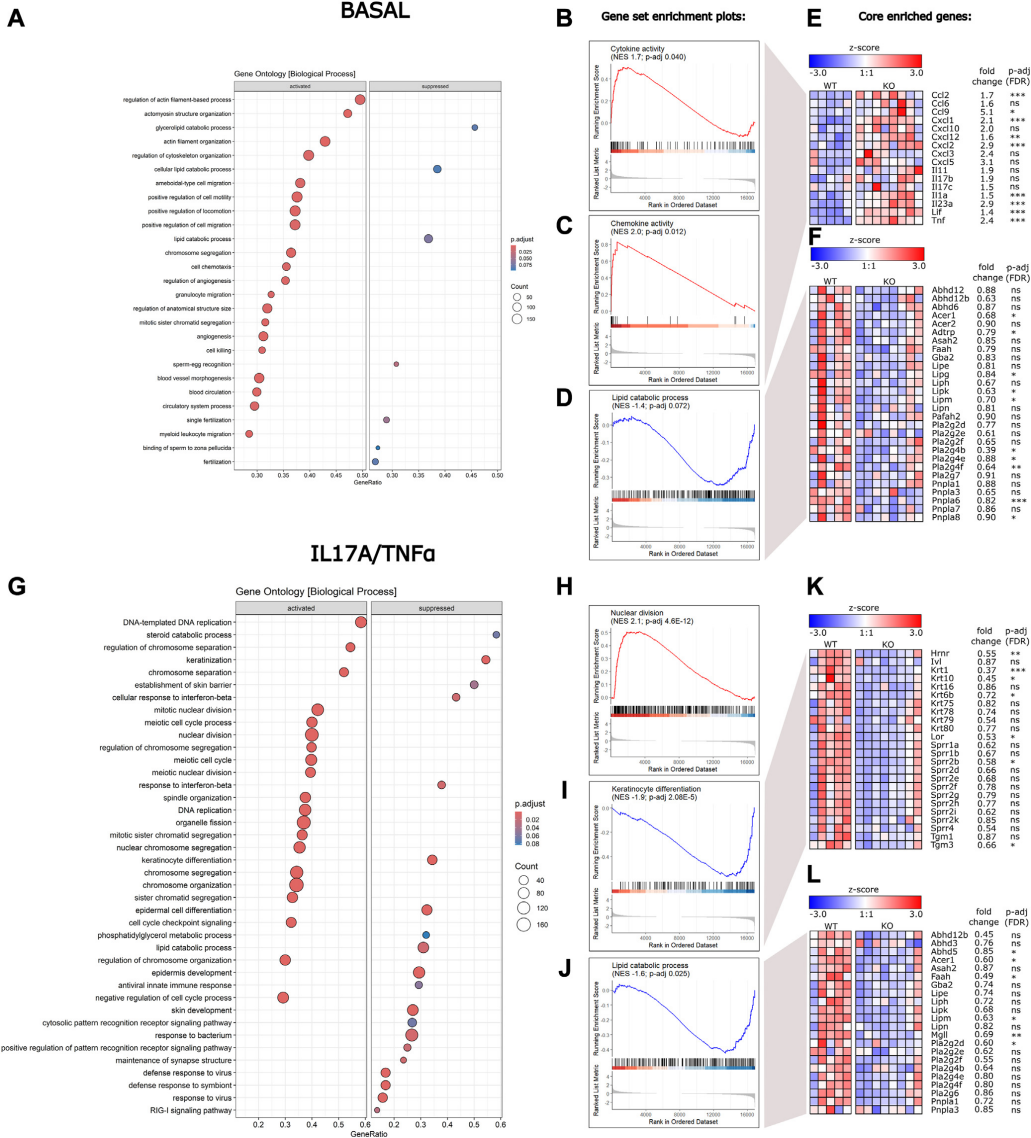
RNA-sequencing of primary WT and *Pla2g4d*-deficient (KO) keratinocytes. Primary keratinocytes were isolated from two-day-old WT (n = 5) and KO (n = 8) neonatal mice and cultured for four days under low CaCl_2_ concentrations (0.06 mM). Subsequently, WT and KO cells were stimulated with IL17A and TNFα (20 ng/ml each) for 24 h. (A) “GO biological process” gene set enrichment analysis (GSEA) comparing KO and WT cells in the absence of cytokines (basal). The top 20 activated and 7 suppressed gene sets are shown. The “gene ratio” (*x*-axis) represents the ratio of core enriched genes (genes before or after the point at which the running enrichment score reaches its maximum or minimum; see B–D and H–J) to the total number of genes in the gene set. The “count” (dot size) reflects the total number of genes in the gene set and the color coding highlights the FDR-adjusted *P* value of the gene set. (B–D) Gene set enrichment plots of GO molecular function gene sets “Cytokine activity” and “chemokine activity,” as well as the GO biological process gene set “lipid catabolic process” comparing KO and WT cells in the absence of IL17A and TNFα (basal). The plots display the GSEA statistics, including the “normalized enrichment score (NES)” and FDR-adjusted *P* value. (E, F) Selected core enriched genes from gene sets shown in (B–D). Differential gene expression between KO and WT cells is visualized in a heat map, displaying the z-score (indicates number of SDs a data point is from the mean of the dataset). Additionally, the fold change (KO/WT) and FDR-adjusted *P* value are shown. (G) “GO biological process” GSEA comparing KO and WT cells in the presence of IL17A and TNFα. The top 20 activated and top 20 suppressed gene sets are shown. (H–J) Gene set enrichment plots of GO biological process gene sets “nuclear division,” “keratinocyte differentiation,” and “lipid catabolic process” comparing KO and WT cells in the presence of IL17A and TNFα. (K, L) Selected core enriched genes from gene sets shown in (I, J). Differential expression between KO and WT cells is visualized in a heat map, displaying the z-score. Additionally, the fold change (KO/WT) and FDR-adjusted *P* value are shown. Statistically significant differences are shown as: ∗*P* < 0.1; ∗∗*P* < 0.01; and ∗∗∗*P* < 0.001. FDR, false discovery rate; PLA2G4, phospholipase A2 group IV.

For the interpretation of gene expression data, we performed gene set enrichment analysis. Under basal culture conditions, gene ontology biological process (GO bp) and GO molecular function (GO mf) terms associated with cell motility, cell migration, cytoskeleton organization as well as cytokine and chemokine activity were significantly activated in KO cells (Fig. 9A and supplemental Fig. S10), while terms associated with lipid catabolic processes were suppressed (Fig. 9A). Selected gene set enrichment plots for “cytokine activity,” “chemokine activity” (GO mf), and “lipid catabolic process” (GO bp) are shown in Figure 9B–D. Here, multiple cytokines and chemokines (*Ccl2*, *Ccl9*, *Cxcl1*, *Cxcl12*, *Cxcl2*, *Il1a*, *Il23a*, *Lif*, *Tnf*) were significantly upregulated, indicating increased inflammation in KO keratinocytes (Fig. 9E). Conversely, expression of genes involved in sphingolipid and glycerolipid metabolism (*Acer1*, *Pla2g4b*, *Pla2g4e*, *Pla2g4f*, *Adtrp*, *Lipg*, *Lipk*, *Lipm*, *Pnpla6*, *Pnpla8*) were suppressed in KO cells under basal conditions (Fig. 9F).

Upon IL17A/TNFα treatment, GO bp terms associated with cell proliferation and nuclear division were upregulated in KO cells (Fig. 9G and H). Suppressed gene sets were associated with epidermis development, keratinization, and keratinocyte differentiation (Fig. 9G and I). Concomitantly, stimulated KO cells expressed significantly reduced levels of early keratinocyte differentiation markers Keratin 1 (*Krt1*), Keratin 10 (*Krt10*), and Keratin 6b (*Krt6b*) (Fig. 9K). Also, several members of the epidermal differentiation complex such as Hornerin (*Hrnr*), loricrin (*Lor*), small proline-rich protein 2B (*Sprr2b*), and transglutaminase 3 (*Tgm3*) were downregulated in KO cells, indicating potential impairment of keratinization and keratinocyte differentiation (Fig. 9K). Similar as observed in the basal state, lipid catabolic processes were again damped (Fig. 9G and J), as indicated by the downregulation of *Abhd5*, *Acer1*, *Faah*, *Lipm*, *Mgll*, and *Pla2g2d*. (Fig. 9L).

## Discussion

The human *PLA2G4D* gene was first identified and cloned in a study investigating differently expressed genes in psoriatic skin (30). Murine *Pla2g4d* was identified, together with *Pla2g4e* and *Pla2g4f*, by performing homology searches using *Pla2g4a* and *Pla2g4c* exons as query sequences (31). In line with our observations, subsequent activity-based assays revealed that PLA2G4D exhibits both phospholipase A1 and A2 activity, but no strict FA and head group preferences (30, 31, 33).

Here, we demonstrate that PLA2G4D mediates the transfer of FAs within phospholipids and acylglycerols (MMAT and DDAT activity), as well as from phospholipids to acylglycerols (PMAT and PDAT activity). Published data already demonstrated that other members of the PLA2G4 family can catalyze the acylation of the alcohol moiety of lysophospholipids and head group acylation of PE and PG (8, 9, 12, 34, 35). Thus, based on structural homology, it is not surprising that PLA2G4D possesses transacylase activity. The enzyme preferentially transferred *sn-*1–linked acyl chains from PC to LPE or from PE to LPC. This positional preference was also observed in PMAT and PDAT reactions, but not in MMAT assays. To our knowledge, PLA2G4D is so far the only identified mammalian enzyme that exhibits PMAT activity, while MMAT, DDAT, and PDAT activity were reported for other enzymes. MMAT and DDAT reactions can be catalyzed by members of the structurally related PNPLA family (5, 36). Recently, DIESL (also known as TMEM68) was identified as a novel TAG synthase, by redistributing acyl chains from phospholipids into the TAG pool, possibly via a PDAT reaction (37). Alternatively, it has been proposed that DIESL possesses acyl-CoA–dependent MGAT and DGAT activity (38). In contrast to mammalian cells, PDAT reactions are well known in plants and yeast (39, 40, 41, 42, 43). Plant PDAT is related to mammalian LCAT and crucially affects carbon flux into seed TAG stores (41).

PLA2G4D showed highest transacylase activity in the presence of MAG. MMAT assays revealed that the enzyme has no strict positional and FA preferences. However, slightly increased activity was observed using 2-oleoyl MAG as substrate. PLA2G4D also acylated 2-arachidonoylglycerol (2-AG) resulting in the formation of arachidonic acid containing DAG. 2-AG is the most abundant endocannabinoid of the body, activating cannabinoid receptors CB1 and CB2 (44, 45). It derives from the degradation of phospholipids (46, 47), and is hydrolyzed by monoglyceride lipase, which disrupts endocannabinoid signaling (48). Transacylation of 2-AG, leading to the formation of DAG, provides an additional plausible mechanism of endocannabinoid inactivation, possibly channeling the endocannabinoid back into phospholipids via DAG kinase (49).

All members of the PLA2G4 family, except PLA2G4C, possess a Ca^2+^-binding C2-domain, which can mediate the targeting of proteins to cell membranes or lipid substrates. Activity assays revealed that PLA2G4D shows activity in the absence and presence of calcium. The addition of CaCl_2_ stimulated phospholipase activity depending on the lipid substrate used, while transacylation reactions were moderately affected. Notably, human PLA2G4D was more sensitive to Ca^2+^-stimulation than the mouse ortholog indicating species differences. Furthermore, we observed that addition of CaCl_2_ during cell fractionation strongly increased the association of PLA2G4D with cellular membranes. Overall, these experiments and the structural features of the enzyme suggest that calcium is involved in the regulation of PLA2G4D function. However, microscopy experiments revealed that the enzyme is primarily cytosolic in the absence and presence of calcimycin and thapsigargin, indicating that an increase in cytosolic Ca^2+^-concentrations is not sufficient to induce its translocation to membranes. To understand the regulation of PLA2G4D in more detail, further studies are required, preferably in a cell system that expresses the enzyme endogenously.

Acylglycerol synthesis in mammalian cells is primarily mediated by acyl-CoA–dependent acyltransferases (50). Notably, however, cells lacking DGAT activity are still capable of synthesizing low amounts of TAG (51, 52), implicating the existence of alternative pathways, which may include transacylation reactions. Our experiments suggest that PLA2G4D can promote the synthesis of acylglycerols. Overexpression of the enzyme in COS7 cells increases the abundance of TAG, particularly favoring the accumulation of PUFA-containing species. The parallel increase in various LPC and LPE species in PLA2G4D-expressing cells suggests that PC/PE act as donor lipids. Considering that PLA2G4D exhibits both phospholipase and transacylase activity, the shift in TAG-FA composition could also be caused by the mobilization of PUFAs from phospholipids, which are subsequently incorporated into TAG via an acyl-CoA–dependent mechanism (53).

The most prominent effect of PLA2G4D overexpression on cellular acylglycerol levels in COS7 cells was observed upon MAG supplementation. Under these conditions, cellular DAG accumulation increased severalfold, consistent with the high MMAT activity of the enzyme. Conversely, total TAG levels were barely affected, likely due to acyl-CoA–dependent reactions mediated by endogenously expressed DGAT. The generated DAG may also be a substrate for DAG kinase generating PA, which is subsequently incorporated into phospholipids via the CDP-DAG or the Kennedy pathway (54). In the presence of DGAT inhibitors, however, PLA2G4D significantly increased both DAG and TAG levels, suggesting that it promotes TAG formation via transacylation reactions. Alternatively, the generated DAG is acylated by other enzymes exhibiting DDAT or PDAT activity. It is important to note that PLA2G4D cannot fully compensate for the lack of DGAT activity, since DGAT-inhibitor treated cells expressing PLA2G4D still exhibit strongly reduced TAG stores in comparison to nontreated cells.

The strong DAG accumulation upon MAG supplementation points toward a role of the enzyme in the metabolization of MAG from extracellular sources. Extracellular MAG predominantly derives from the degradation of TAG-rich lipoproteins by lipoprotein lipase, which cleaves TAG at *sn*-1 and *sn*-3 positions, resulting in the formation of 2-MAG and FFA (55, 56). In principle, MAG generated from extracellular sources can be degraded by MAG lipases, re-esterified by acyl-CoA–dependent mechanisms, or re-esterified by acyl-CoA–independent mechanisms involving PLA2G4D. The disposition of lipoprotein-derived MAG is poorly investigated. An in vivo study comparing the metabolization of radiolabeled triolein and a nondegradable 2-ether analog of triolein suggested that MAG generated by lipoprotein lipase predominantly undergoes re-esterification in heart and liver, while in adipose and muscle MAG is partially degraded into glycerol and FA (57). A second important source of MAG originates from the hydrolysis of dietary lipids by pancreatic lipase in the intestinal lumen. According to our current knowledge, epithelial cells of the intestine take up dietary MAG and FFA, and use them for chylomicron production, mainly via the acyl-CoA–dependent MGAT/DGAT pathways (58). A further detailed study of PLA2G4D expression and regulation will reveal whether the enzyme contributes to acylglycerol synthesis in specific tissues or cell types. Our observations suggest that *Pla2g4d* mRNA is broadly expressed in tissues of ectodermal origin including skin, testis, and brain. Within the gastrointestinal tract, high expression was observed only in the stomach.

It is important to note that experiments in COS7 cells ectopically expressing PLA2G4D do not necessarily reflect the physiological function of PLA2G4D. These cells as well as other commonly used cell lines show no or poor endogenous expression. PLA2G4D is found in keratinocytes and potentially in other specialized cell types such as mast cells (32, 59). It is upregulated in inflammatory skin disease by proinflammatory cytokines indicating a role in cell signaling pathways. To obtain further insights into its physiological function, we studied lipidomic and transcriptional alterations in keratinocytes lacking PLA2G4D. Notably, under basal conditions, we observed no significant lipidomic changes, suggesting that lack of PLA2G4D activity is compensated by other enzymes. In the presence of cytokines that stimulate *Pla2g4d* mRNA expression, however, we found substantial lipidomic changes implicating that PLA2G4D is involved in the remodeling of the lipidome under inflammatory conditions. The observed increased abundance of SM and CER cannot be explained by the in vitro activity of PLA2G4D while increased levels of PC, PE, etherPC, and etherPE indicate that the enzyme accepts phospholipids and ether phospholipids as substrate. Although total DAG and TAG levels were unchanged in KO cells, we observed the accumulation of saturated and monounsaturated subspecies, indicating that PLA2G4D deficiency moderately affects acylglycerol metabolism in keratinocytes under the applied conditions.

The observed lipidomic changes in KO keratinocytes may be caused directly by the lack of PLA2G4D activity or indirectly by dysregulated signaling pathways altering gene expression and/or posttranslational events. Analysis of transcriptional changes revealed that *Pla2g4d* deficiency is associated with the dysregulation of multiple genes affecting proliferation, differentiation, inflammation, and lipid metabolism. Specifically, the downregulation of lipid catabolic enzymes suggests that lipidomic changes may at least be partially caused by the dysregulation of cell signaling pathways.

In conclusion, our observations suggest that PLA2G4D exhibits hydrolase and transacylase activity using both phospholipids and acylglycerols as substrates. This broad substrate specificity allows the degradation of glycerolipids and their synthesis via acyl-CoA–independent mechanisms. PLA2G4D is expressed in keratinocytes and activated under inflammatory conditions, where it modulates metabolic and signaling pathways in a complex manner.

## Data availability

The data that support the findings of this study are listed in the article and are available from the corresponding author upon reasonable request. Contact: Robert Zimmermann, robert.zimmermann@uni-graz.at; Institute of Molecular Biosciences, University of Graz, Heinrichstraße 31, 8010 Graz, Austria.

## Supplemental data

This article contains supplemental data.

## Conflict of interest

The authors declare that they have no conflicts of interest with the contents of this article.
