# Supplementary material for: Phospholipase A2 group IVD mediates the transacylation of glycerophospholipids and acylglycerols
Source: J Lipid Res. 2024 Oct 25;65(12):100685. doi: 10.1016/j.jlr.2024.100685 (PMC11621493; doi:10.1016/j.jlr.2024.100685)
Supplement: Supplemental Figs. S1–S10 and Tables S1–S3 [file mmc1.docx]

#

# SUPPLEMENTAL INFORMATION

# Phospholipase A2 group IVD mediates the transacylation of glycerophospholipids and acylglycerols

Johannes Breithofer^1^*, Dominik Bulfon^1^*, Nermeen Fawzy^1^, Martin Tischitz^1^, Clara Zitta^1^, Lennart Hartig^1^, Gernot F. Grabner^2^, Anita Pirchheim^2^, Hubert Hackl^3^, Ulrike Taschler^1^, Achim Lass^1,5,6^, Carmen Tam-Amersdorfer^4^, Herbert Strobl^4^, Dagmar Kratky^2,5^, and Robert Zimmermann^1,5,6#^


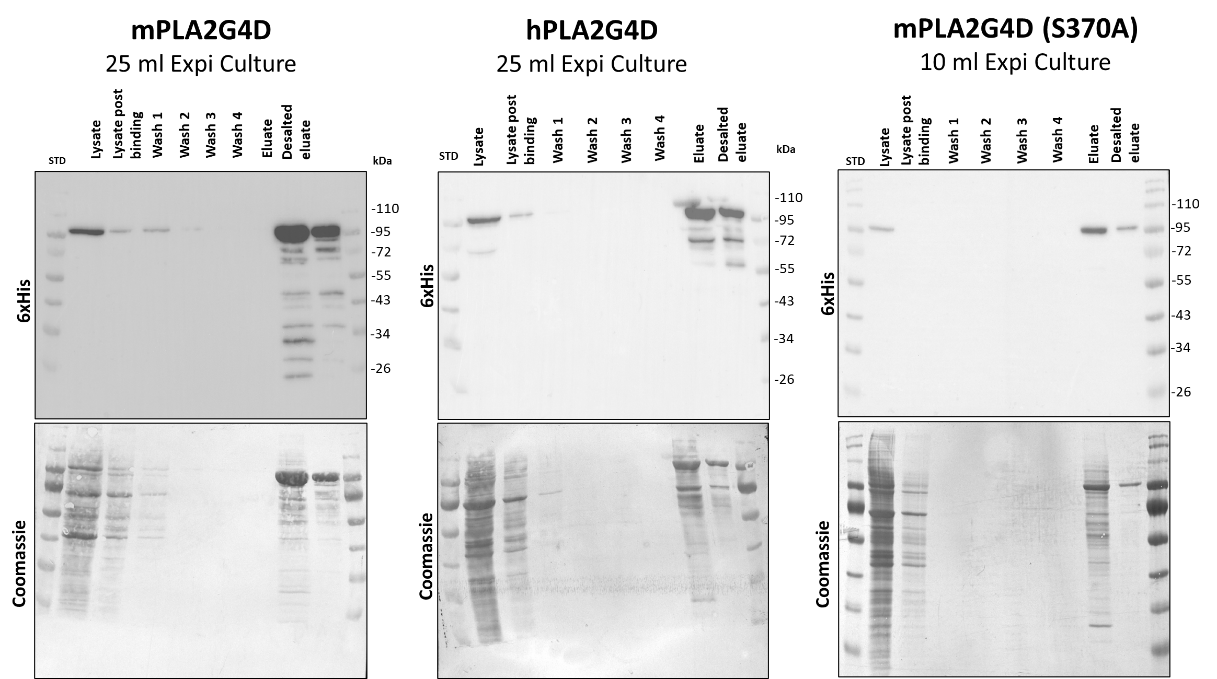


**A**

**B**

**C**

## Figure S1: Partial purification of PLA2G4D.

**(A-C)** Partial purification of 6xHis tagged mPLA2G4D (expressed in 25 ml Expi293F culture), hPLA2G4D (expressed in 25 ml Expi293F culture), and mPLA2G4D S370A (expressed in 10 ml Expi293F culture) using the TALON Metal affinity resin. Protein fractions (5µl) were analyzed by Western blotting using an anti-6xHIS antibody and Coomassie blue staining.

**
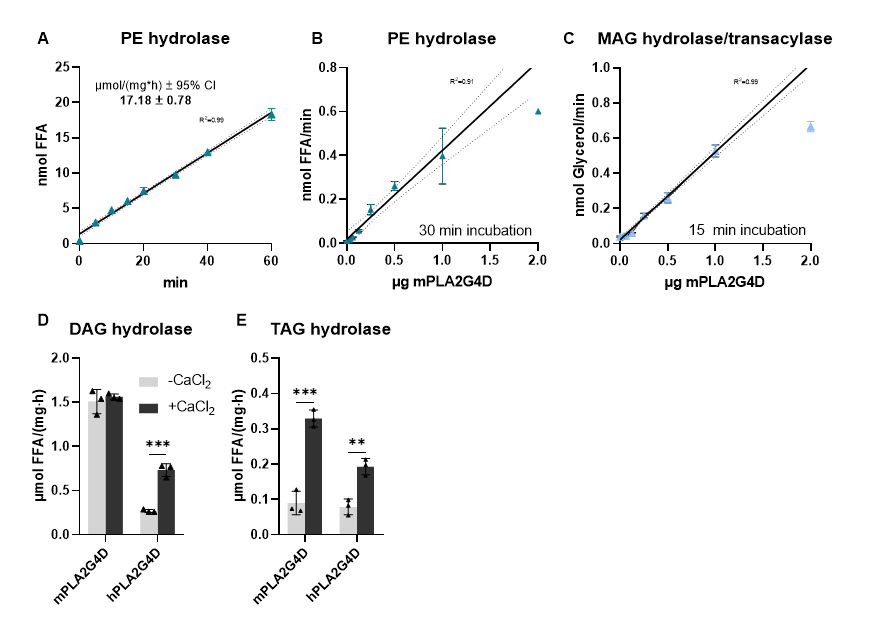
**

## Figure S2: Time- and dose-dependent phospholipase and transacylase as well as DAG and TAG hydrolase activities of PLA2G4D.

**(A)** Time-dependent formation of FFAs in the presence of mPLA2G4D using 18:1 PE as substrate. The assay was performed in the presence of 1 mM CaCl_2_ (n=3). The release of FFAs was quantified using commercial kits. Linear regression was used to calculate the specific PE hydrolase activity of the enzyme.

**(B)** 18:1 PE hydrolase activity of mPLA2G4D at increasing protein concentrations (0-2 µg) in the presence of 1 mM CaCl_2_ (n=2). The assay was incubated for 30 min. Linear regression was used to show linearity between 0 and 1 µg of protein.

**(C)** *rac*-18:1 MAG hydrolase/transacylase activity of mPLA2G4D at increasing protein concentrations (0-2 µg, n=2). The assay was incubated for 15 min. Glycerol release was determined using commercial colorimetric kits. Linear regression was used to show linearity between 0 and 1 µg of protein.

The dotted line in figure (A-C) indicates the 95% confidence interval (CI), and R^2^ shows the coefficient of determination. Enzyme activity assays in (A-C) were carried out by incubating partially purified mPLA2G4D with 20 µl of lipid substrate (1 mM) in PBS (pH 7.4) containing 2% BSA (FA free).

**(D&E)** Radiolabeled DAG and TAG hydrolase activity assay with m- and hPLA2G4D in the absence and presence of 1 mM CaCl_2_ (n=3). 1 µg of partially purified protein diluted in 25 µl enzyme storage buffer (50 mM Tris-HCl pH 7.4, 20 µM DTT, 250 mM sucrose) was incubated with 25 µl substrate containing ^3^H-labeled sn-1,3 18:1 DAG (0.32 mM) or 18:1 TAG (0.3 mM) emulsified with 45 and 50 µM PC/PI (3:1), respectively, in 100 mM potassium-phosphate buffer (pH 7.4) supplemented with 5% BSA (FA free). Free fatty acid release was determined by liquid scintillation.

Data are presented as mean ± SD. Statistical comparison in (D and E) was performed with multiple unpaired two-tailed Student’s t-test followed by Bonferroni post hoc analysis. Statistically significant differences are shown as: *p < 0.05; **p < 0.01; ***p < 0.001.


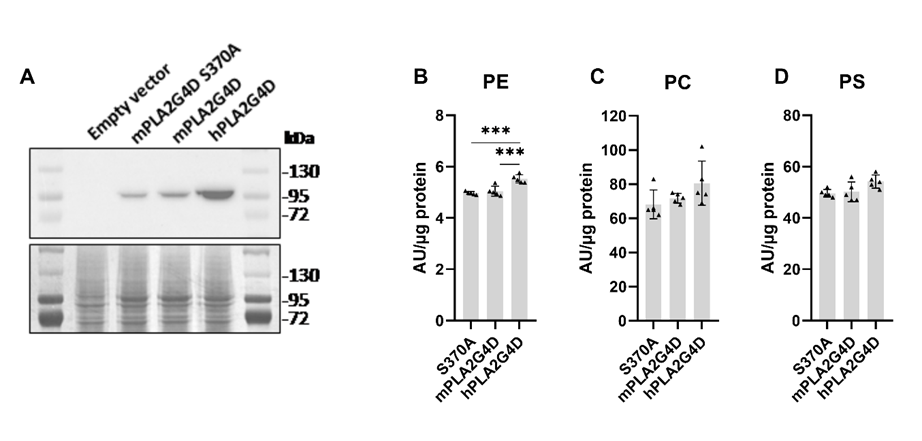


## Figure S3: PLA2G4D overexpression alters cellular acylglycerol and phospholipid profiles.

**(A)** Western blot showing the overexpression of mPLA2G4D S370A, mPLA2G4D, and hPLA2G4D in COS7 cells. The proteins were detected using an antibody against a 6xHis tag. Coomassie blue staining was used as loading control.

**(B-D)** Total PE, PC, and PS levels of COS7 cells expressing m- and hPLA2G4D or the mS370A mutant 48 h post-transfection. Total PE, PC, and PS levels were calculated based on the sum of signals from all subspecies analyzed with HPLC-MS (n=5). Data is presented as mean ± SD. Statistical comparison was performed with one-way ANOVA followed by Bonferroni post hoc analysis. Statistically significant differences are shown as: **p* < 0.05; ***p* < 0.01; ****p* < 0.001.

## Figure S4: HPLC-MS analysis of phospholipids and acylglycerols in COS7 cells overexpressing PLA2G4D.

**(A-G)** Lipid subspecies of PE, PC, PS, MAG, DAG, and TAG classes in COS7 cells expressing mPLA2G4D or the mS370A mutant 48 h post-transfection.

**(H-N)** Lipid subspecies of PE, PC, PS, MAG, DAG, and TAG classes in COS7 cells expressing hPLA2G4D or the mS370A mutant 48 h post-transfection.

Lipids were analyzed with HPLC-MS and are labeled according to the number of C-atoms in fatty acids and the total number of double bonds. This nomenclature can overlap among acylglycerol subspecies despite having different acyl-chain composition. TAGs indicated in ‘**bold**’ highlight arachidonic acid (20:4)- and/or docosahexaenoic acid (22:6)-containing subspecies as determined in the multiple reaction monitoring during HPLC-MS analysis. Data is presented as mean ± SD (n=5). Statistical comparison was performed with multiple unpaired two-tailed Student’s *t*-test followed by Bonferroni post hoc analysis. Statistically significant differences are shown as: **p* < 0.05; ***p* < 0.01; ****p* < 0.001.

## Figure S5: Incorporation of radiolabeled MAG or fatty acids into lipid pools of COS7 cells overexpressing PLA2G4D.

**(A&B)** Incorporation of radioactivity into polar lipids (PL), MAG, DAG, and TAG pools of control and mPLA2G4D-overexpressing COS7 cells loaded with 300 µM *rac*-18:1 MAG and ^14^C-glycerol-backbone-labeled 2-18:1 MAG as tracer (A), or 300 µM ^3^H-labeled oleic acid (B). Cells were incubated with the radioactive tracers for 4h. Subsequently, cells were extensively washed with PBS and lipids were extracted hexane/isopropanol (v/v, 3:2). The experiments were performed 48 h post transfection.

Data are presented as mean ± SD (n=3) and statistical comparison was performed with an unpaired two-tailed Student’s *t*-test. Statistically significant differences are shown as: **p* < 0.05; ***p* < 0.01; ****p* < 0.001.


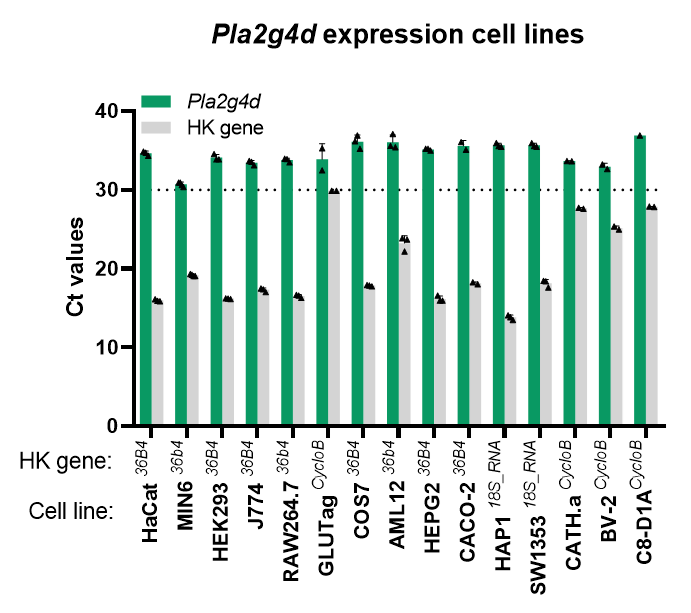


## Figure S6: *Pla2g4d* mRNA expression in frequently used cell lines.

RT-PCR data (Ct values) of *Pla2g4d*- and house-keeping (HK) gene expression in various cell lines. Ct values above 30 indicate no or negligible expression for *Pla2g4d*. *Pla2g4d* primers were designed to target murine, human, and monkey *Pla2g4d* mRNA.


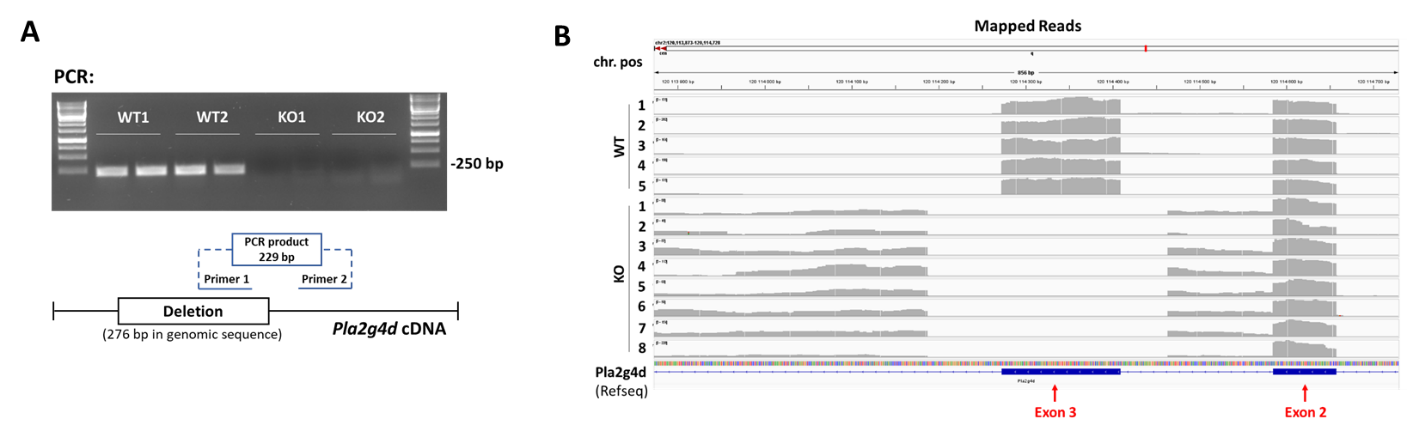


## Figure S7: Confirmation of CRISPR/Cas9-generated deletion in the *Pla2g4d* gene.

**(A)** Agarose gel electrophoresis of a RT-PCR reaction using cDNA from primary WT and *Pla2g4d*-deficient keratinocytes stimulated with IL17A/TNFα (20 ng/ml each) as template. One primer binds within the deletion resulting in a missing PCR product in KO samples.

**(B)** RNA sequencing reads (grey bars) generated from RNA of five wild-type (WT) and eight *Pla2g4d-*deficient (KO) primary keratinocytes stimulated with IL17A and TNFα (20 ng/ml each). Reads were mapped to the *mm39* mouse reference genome. The figure shows exon 3, where the CRISPR-generated 276 bp deletion is located, and exon 2 of the Pla2g4d gene. In KO cells, no reads are mapped in exon 3.


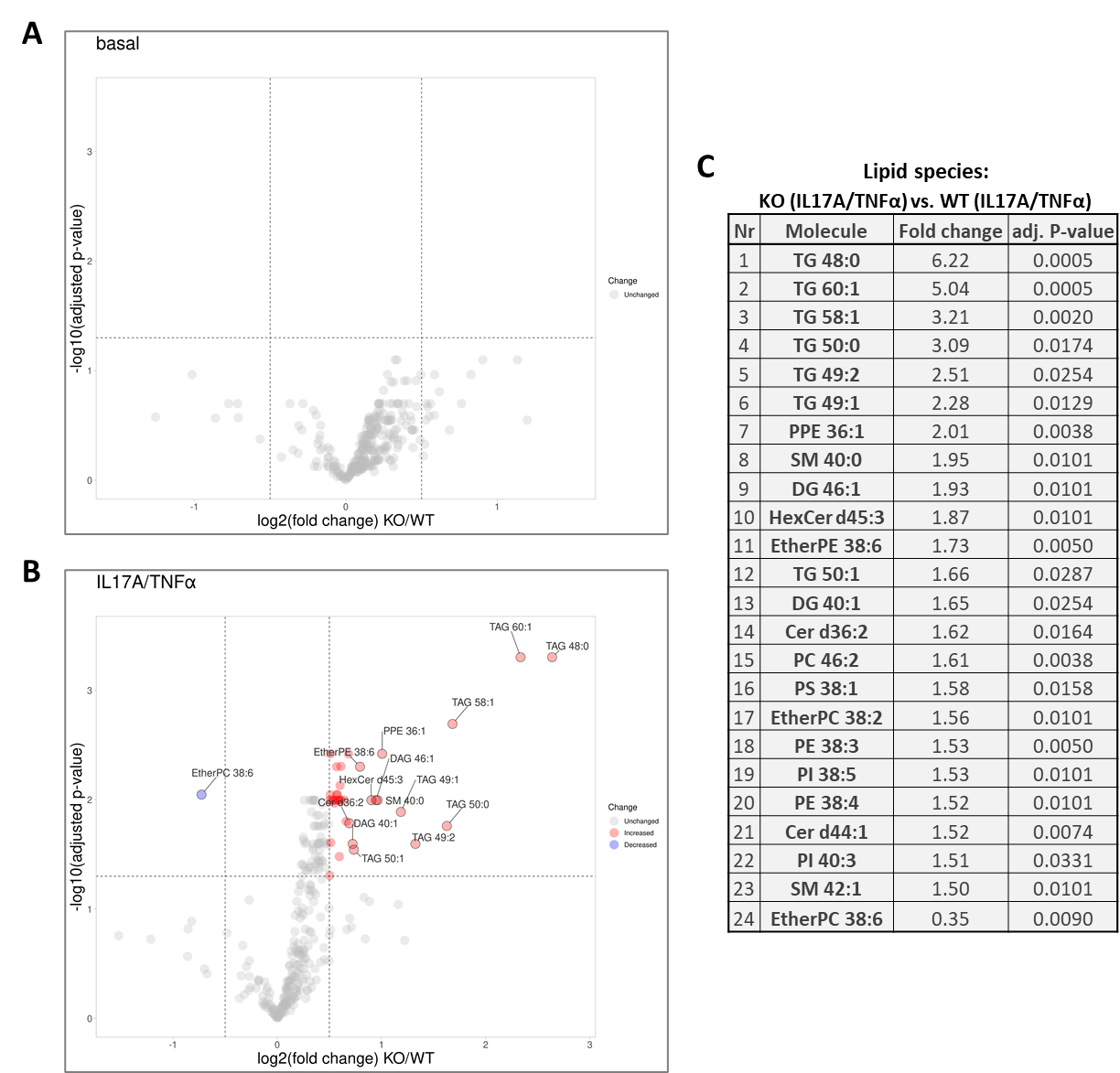


## Figure S8*:* Most regulated molecular lipid species between primary wild-type (WT) and *Pla2g4d*-deficient (KO) keratinocytes in the absence and presence of cytokines.

Primary keratinocytes were isolated from two day old WT (n=5) and KO (n=8) neonatal mice and cultured for four days under low CaCl_2_ concentrations (0.06 mM). Subsequently, WT and KO cells were stimulated with IL17A and TNFα (20 ng/ml each) for 24 h.

**(A&B)** Volcano plots showing the log_2_(fold change) and -log10(adjusted *p*-value) of 296 analyzed molecular lipid species between KO and WT keratinocytes cultured in the absence (A) and presence (B) of cytokines. Molecular lipid species with a fold decrease or increase > 1.5 and adj: *p*-value < 0.05 were considered as significantly changed.

**(C)** Fold change and adjusted *p*-value of significantly changed lipid species between KO and WT keratinocytes in the presence of cytokines.

Statistical comparisons were performed with multiple unpaired two-tailed Student’s *t*-test with correction for multiple comparisons using the False Discovery Rate (FDR).


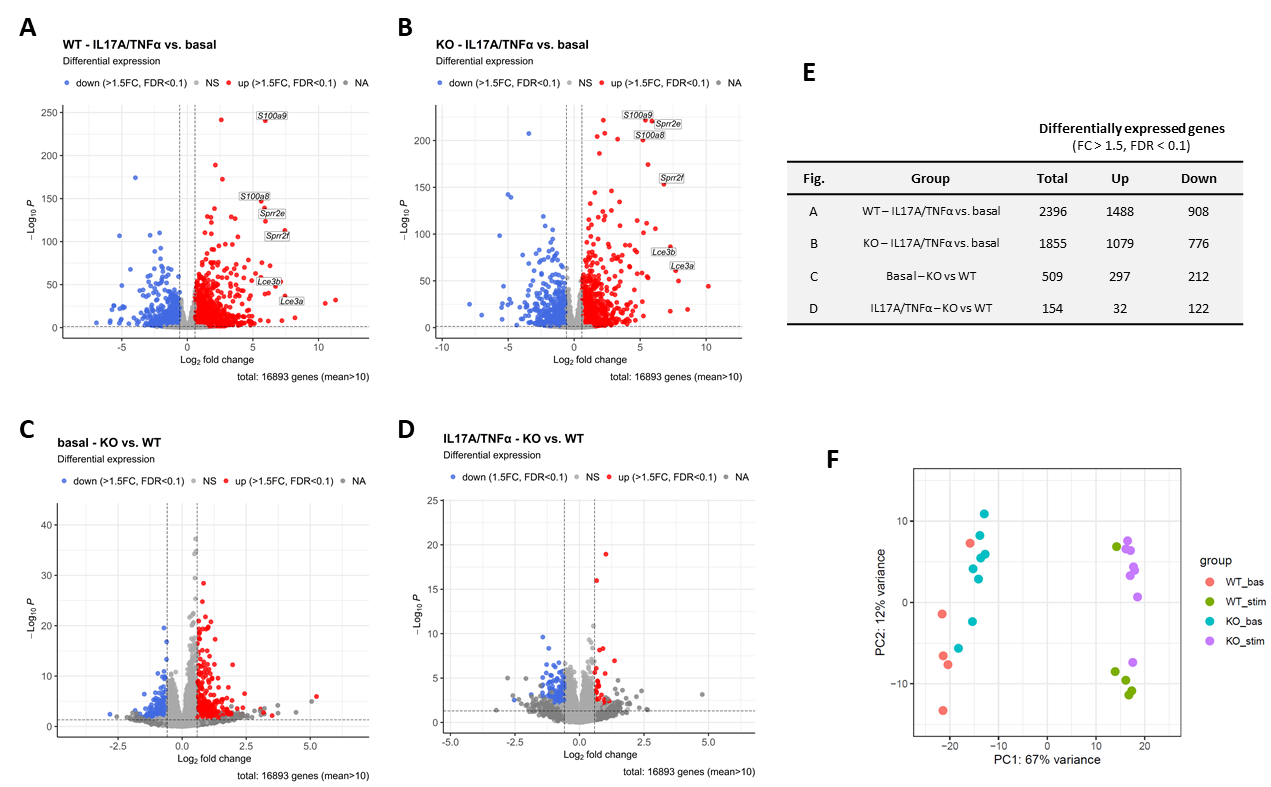


## Figure S9 Differential gene expression in primary wild-type (WT) and *Pla2g4d*-deficient (KO) keratinocytes:

Primary keratinocytes were isolated from two days old WT (n=5) and KO (n=8) neonatal mice and cultured for four days under low CaCl_2_ concentrations (0.06 mM). Subsequently, WT and KO cells were stimulated with IL17A and TNFα (20 ng/ml each) for 24 h.

**(A-D)** Volcano plots showing the log_2_(fold change) and -log_10_(*p*-value) of 16893 detected RNA transcripts with average expression across all samples >10 in primary kerationcytes. The effects of cytokine treatment on both WT (A) and KO (B) cells, as well as the changes between genotypes under basal (C) culture conditions and cytokine stimulation (D) are shown. Genes with a fold decrease or increase >1.5 and FDR adjusted *p*-value <0.1 were considered as differentially expressed.

**(E)** Number of differentially expressed genes under conditions shown in (A-D).

**(F)** Principal component analysis (PCA) of RNA sequencing data shown in (A-D). PCA was calculated using the top 5000 genes, excluding those encoded by the X and Y chromosomes due to the experiment not considering the gender of the isolated cells.


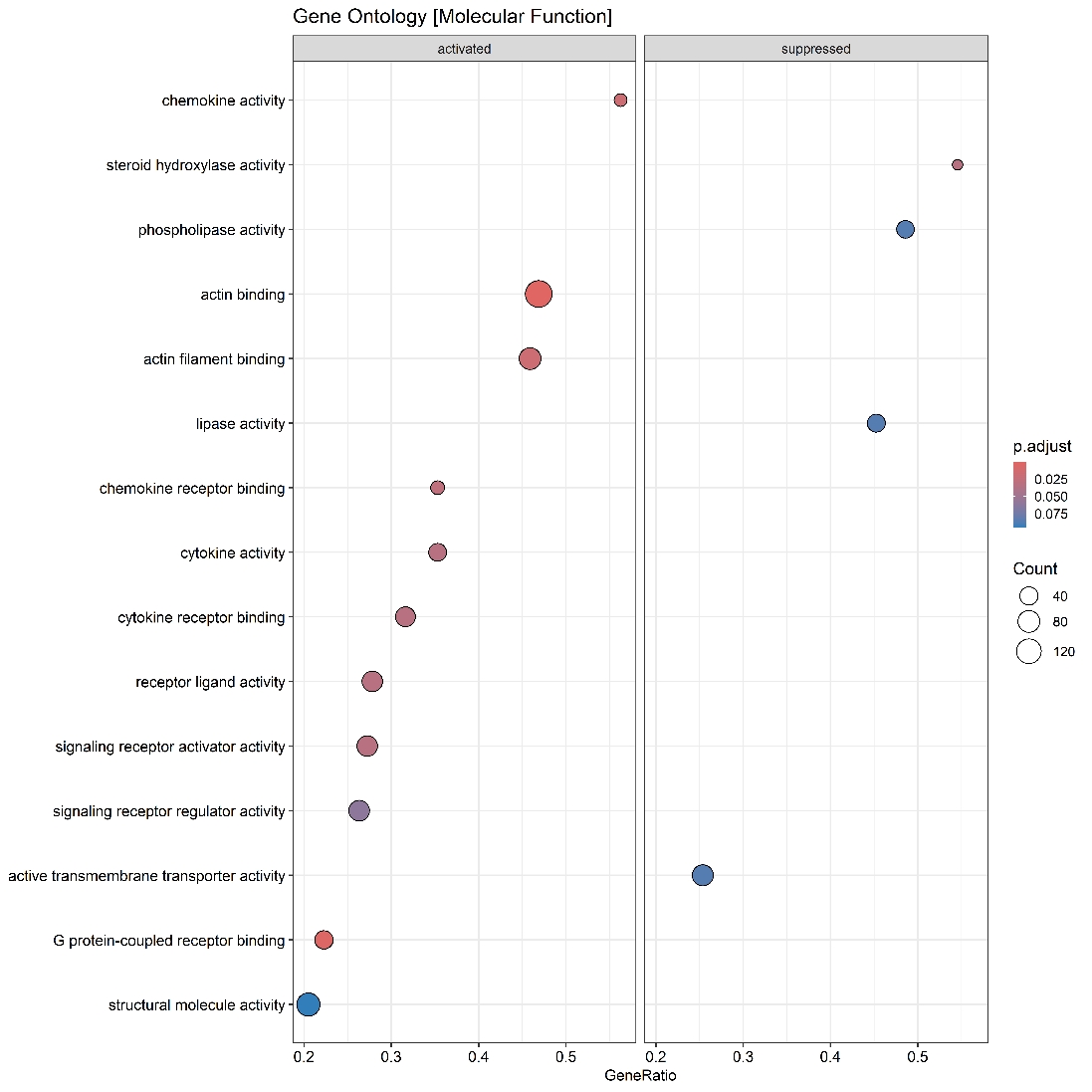


## Figure S10: “GO molecular function” Gene set enrichment analysis (GSEA) between primary *Pla2g4d*-deficient (KO) and wild-type keratinocytes in the absence of IL17A and TNFα (basal).

The 11 activated and 4 suppressed gene sets are shown. The “gene ratio” (x-axis) represents the ratio of core enriched genes (genes before or after the point at which the running enrichment score reaches its maximum or minimum) to the total number of genes in the gene set. The “count” (dot size) represents the total number of genes in the gene set and the color coding highlights the FDR-adjusted *p*-value of the gene set.

## Table S1: Lipid substrates

| **Phospholipid** | **Company** | **Catalog number** |
| --- | --- | --- |
| 18:1 Phosphatidylcholine | Avanti Polar Lipids | Cat#850375P |
| 18:1 Phosphatidylethanolamine | Avanti Polar Lipids | Cat#850725P |
| 18:1 Phosphatidylglycerol | Avanti Polar Lipids | Cat#840475P |
| 18:1 Phosphatidylserine | Avanti Polar Lipids | Cat#840035P |
| 18:1 Phosphatidic acid | Avanti Polar Lipids | Cat#840875P |
| 18:1 Phosphatidylinositol | Avanti Polar Lipids | Cat#850155P |
| *sn*-1-18:1 Lyso-phosphatidylcholine | Avanti Polar Lipids | Cat#845875P |
| *sn*-1-18:1 Lyso-phosphatidylethanolamine | Avanti Polar Lipids | Cat#846725P |
| *sn*-1-18:1 Lyso-phosphatidylglycerol | Avanti Polar Lipids | Cat#858125P |
| *sn*-1-18:1 Lyso-phosphatidylserine | Avanti Polar Lipids | Cat#858143P |
| *sn*-1-18:1 Lyso-phosphatic acid | Avanti Polar Lipids | Cat#857230P |
| *sn*-1-16:0-*sn*-2-20:4 Phosphatidylcholine | Avanti Polar Lipids | Cat#850459 |
| *sn*-1-16:0-*sn*-2-20:4 Phosphatidylethanolamine | Avanti Polar Lipids | Cat#850759 |
| *rac*-18:1 Monoacylglycerol | Sigma Aldrich | Cat#M7765 |
| *rac*-18:1 Diacylglycerol | Sigma Aldrich | Cat#D8894 |
| *sn-*1,3-18:1 Diacylglycerol | Sigma Aldrich | Cat#D3627 |
| 2-20:4 Monoacylglycerol | Avanti Polar Lipids | Cat#870450O |
| *2-*18:1 Monoacylglycerol | Larodan | Cat#31-1812 |

## Table S2: List of antibodies

| **Antibodies** | **Company** | **Catalog number** |
| --- | --- | --- |
| Anti-6X His tag® antibody | Abcam | Cat#ab18184 |
| [Rabbit Anti-GAPDH Monoclonal Antibody](https://scicrunch.org/resources/data/record/nif-0000-07730-1/AB_561053/resolver?q=2118S%20Gapdh&l=2118S%20Gapdh&i=2068511) | Cell Signaling Technology | Cat#2118 |
| [Pan-Cadherin Antibody](https://scicrunch.org/resources/data/record/nif-0000-07730-1/AB_2158565/resolver?q=pan-%20cadherin%20%234068&l=pan-%20cadherin%20%234068&i=880868) | Cell Signaling Technology | Cat#4068 |
| [Calnexin (C5C9) Rabbit mAb](https://scicrunch.org/resources/data/record/nif-0000-07730-1/AB_2228381/resolver?q=%232679%20Cell%20signaling&l=%232679%20Cell%20signaling&i=366142) | Cell Signaling Technology | Cat#2679 |
| Anti-SDHA antibody | Abcam | Cat#ab14715 |
| Anti-Lamp1 | Cell Signaling Technology | Cat#3243 |
| [Goat Anti-Rabbit IgG Antibody (H+L), Peroxidase](https://scicrunch.org/resources/data/record/nif-0000-07730-1/AB_2916034/resolver?q=PI-1000%3B%20Vector%20Laboratories&l=PI-1000%3B%20Vector%20Laboratories&i=2851490) | Vector Laboratories | Cat#PI-1000-1 |
| [Sheep Anti-Mouse IgG ECL Antibody, HRP Conjugated](https://scicrunch.org/resources/data/record/nif-0000-07730-1/AB_772193/resolver?q=NA9310&l=NA9310&i=2127528) | Cytiva | Cat#NA9310-1ml |
| [Anti-mouse IgG (H+L), F(ab)2 Fragment (Alexa Fluor 594 Conjugate)](https://scicrunch.org/resources/data/record/nif-0000-07730-1/AB_2714182/resolver?q=Cell%20signaling%20%238890&l=Cell%20signaling%20%238890&i=2649638) | Cell Signaling Technology | Cat#8890 |

## Table S3: RT-PCR primers

| **Name** | **5’-3’ Sequence** |  |
| --- | --- | --- |
| mPla2g4d_fwd | GTCTTCCTTCCTCCTTCCTG |  |
| mPla2g4d_rev | ATCTGACAAAACCCAAACCC |  |
| hPla2g4d_fwd | GCTGACCTGTTGAGTGAGGC |  |
| hPla2g4d_rev | TCGGTGAGCGTCTTGGTCTTA |  |
| mkPla2g4d_fwd | CCCGTACGTGATCCTACAGC |  |
| mkPla2g4d_rev | ATGCACATCCAGGCATGACA |  |
| 36B4_fwd | GCTTCATTGTGGGAGCAGACA |  |
| 36B4_rev | CATGGTGTTCTTGCCCATCAG |  |
| Cyclo_B_fwd | GGCTCCGTCGTCTTCCTTTT |  |
| Cyclo_B_rev | ACTCGTCCTACAGATTCATCTCC |  |
| 18SrRNA_fwd | GTAACCCGTTGAACCCCATT |  |
| 18SrRNA_rev | CCATCCAATCGGTAGTAGCG |  |
